# Identification of Upstream Transcriptional Regulators of Ischemic Cardiomyopathy Using Cardiac RNA-Seq Meta-Analysis

**DOI:** 10.3390/ijms21103472

**Published:** 2020-05-14

**Authors:** Ahmad Alimadadi, Sachin Aryal, Ishan Manandhar, Bina Joe, Xi Cheng

**Affiliations:** 1 Center for Hypertension and Precision Medicine, Program in Physiological Genomics, Department of Physiology and Pharmacology, University of Toledo College of Medicine and Life Sciences, Toledo, OH 43614, USA; ahmad.alimadadi@rockets.utoledo.edu (A.A.); sachin.aryal@rockets.utoledo.edu (S.A.); ishan.manandhar@rockets.utoledo.edu (I.M.); bina.joe@utoledo.edu (B.J.); 2 Bioinformatics Program, University of Toledo College of Medicine and Life Sciences, Toledo, OH 43614, USA

**Keywords:** ischemic cardiomyopathy, heart failure, transcriptional regulators, meta-analysis, RNA-seq

## Abstract

Ischemic cardiomyopathy (ICM), characterized by pre-existing myocardial infarction or severe coronary artery disease, is the major cause of heart failure (HF). Identification of novel transcriptional regulators in ischemic HF can provide important biomarkers for developing new diagnostic and therapeutic strategies. In this study, we used four RNA-seq datasets from four different studies, including 41 ICM and 42 non-failing control (NF) samples of human left ventricle tissues, to perform the first RNA-seq meta-analysis in the field of clinical ICM, in order to identify important transcriptional regulators and their targeted genes involved in ICM. Our meta-analysis identified 911 differentially expressed genes (DEGs) with 582 downregulated and 329 upregulated. Interestingly, 54 new DEGs were detected only by meta-analysis but not in individual datasets. Upstream regulator analysis through Ingenuity Pathway Analysis (IPA) identified three key transcriptional regulators. *TBX5* was identified as the only inhibited regulator (*z*-score = −2.89). *F2R* and *SFRP4* were identified as the activated regulators (*z*-scores = 2.56 and 2.00, respectively). Multiple downstream genes regulated by *TBX5*, *F2R*, and *SFRP4* were involved in ICM-related diseases such as HF and arrhythmia. Overall, our study is the first to perform an RNA-seq meta-analysis for clinical ICM and provides robust candidate genes, including three key transcriptional regulators, for future diagnostic and therapeutic applications in ischemic heart failure.

## 1. Introduction

Heart failure (HF), leading to considerable mortality and health care costs, is a critical health problem, especially among the people aged ≥65, around the world [1]. Characterized by pre-existing myocardial infarction, hibernating myocardium or severe coronary artery disease, ischemic cardiomyopathy (ICM) accounted for more than 60% of systolic HF cases in industrialized countries [2]. Mild or severe repeated injuries to left ventricle are common in patients with ICM, resulting in cardiac remodeling, chronic myocardial dysfunction and eventual HF [3].

Dysregulated transcriptional hubs, such as transcription factors, non-coding RNAs and chromatin regulators, and their downstream gene expression signatures are representative of genomic mechanisms underlying disease processes. Therefore, detecting such genomic signatures aids in the development of new diagnostic and therapeutic approaches. A few such independent studies have reported unique gene expression signatures eventually leading to cardiovascular diseases such as HF [4,5,6,7].

RNA-seq studies play an important role in understanding transcriptional regulation of cardiovascular diseases [8]. Since different sample and data processing strategies among different RNA-seq studies could generate contradicting results, meta-analysis has been widely used to overcome inconsistent findings among different studies [9,10]. In this study, we used four RNA-seq datasets from four different RNA-seq studies, including 41 ICM and 42 non-failing control (NF) samples of human left ventricle tissues, to perform the first RNA-seq meta-analysis for identifying key transcriptional regulators involved in clinical ICM.

## 2. Results

### 2.1. RNA-Seq Datasets

Four RNA-seq datasets (GSE116250 [11], GSE120852 [12], GSE46224 [13] and GSE48166 [14]), including 41 ICM and 42 NF samples, were included and analyzed in this study (Table 1). The information of each dataset was summarized in Table 1.

### 2.2. Differentially Expressed Genes

**Figure 1:**
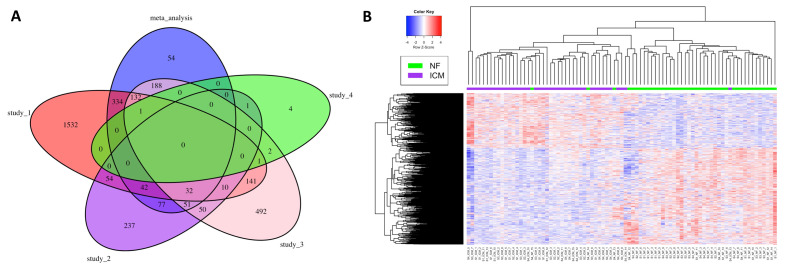
Venn diagram and heatmap summarizing meta-analysis identified DEGs. (**A**) Venn diagram summarizing common DEGs among the meta-analysis and the individual studies. (**B**) Heatmap of the DEGs identified by the meta-analysis. S1, S2, S3, S4: Study_1, Study_2, Study_3, Study_4 (Table 1).

Expression levels of 58,884 coding and non-coding genes based on Homo_sapiens.GRCh38.96.gtf were quantified in this study. Nine hundred and eleven (911) differentially expressed genes (DEGs), including 582 downregulated and 329 upregulated genes, were identified in meta-analysis (Supplementary Table S1). The top 50 DEGs ordered by *p*-value are presented in Table 2. The number of common DEGs among the meta-analysis and the analyses of four individual studies are presented using Venn diagram (Figure 1A). A considerable number of DEGs were not consistent among different RNA-seq studies in terms of statistical significance (Figure 1A). Interestingly, 54 new DEGs were identified in the meta-analysis but not in the individual datasets (Figure 1A, Table 3). No common genes were identified as statistically significant among the meta-analysis and all four individual analyses (Figure 1A). However, a heatmap of all meta-analysis identified DEGs showed consistent patterns of up- or down-regulated DEGs among the samples across different studies (Figure 1B).

### 2.3. Toxicity Pathway Analysis

**Figure 2:**
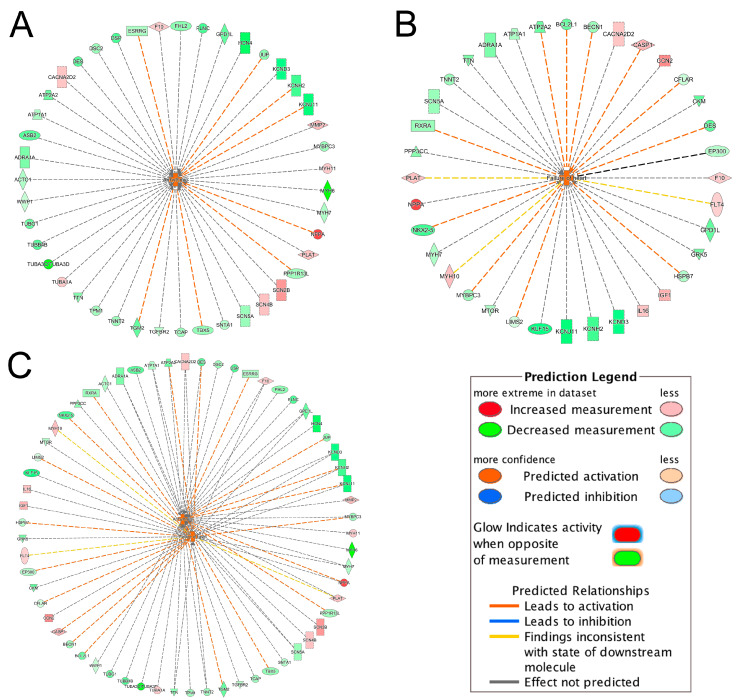
Interactions of significantly activated toxicity pathways with their associated DEGs. (**A**) arrhythmia; (**B**) failure of heart; (**C**) integrated toxicity pathways. For example, in Figure 2A, the gene *TBX5* was downregulated as indicated by the green color and the downregulation of *TBX5* further promoted (indicated by the orange dash line) the activation of arrhythmia, as indicated by the orange color. For other indicators, please refer to the Prediction Legend.

The IPA-Tox analysis identified 232 significant toxicity pathways (*p*-value < 0.05). Activation *z*-score > 2 was considered as significantly activated. Only two pathways, arrhythmia (*z*-score = 2.81) and failure of heart (*z*-score = 2.41), were marked with significant activation status. Interactions of these two pathways with their associated DEGs are shown in Figure 2A,B. To identify shared DEGs between these two pathways, an integrated network was generated (Figure 2C). Seventeen genes out of 62 DEGs were shared between the two pathways and interestingly, most of them were downregulated in the ICM group (Figure 2C, Table 4). Among the common DEGs between failure of heart and arrhythmia, *DES* and *TNNT2* were among the top 50 DEGs (Figure 2C, Table 2). *ATP1A1* was among the DEGs that were identified only in meta-analysis but not in the four individual datasets (Figure 2C, Table 3).

### 2.4. Canonical Pathway Analysis

**Figure 3:**
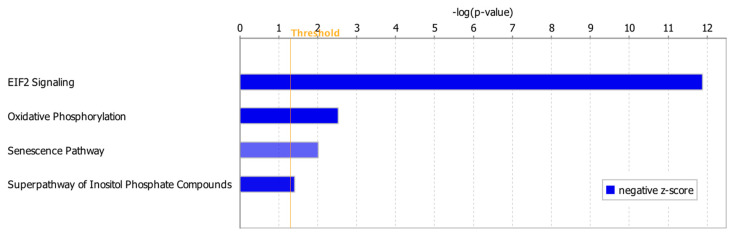
Inhibited canonical pathways in the ICM group. All the inhibited pathways had *z*-scores smaller than −2.0 (Supplementary Table S2).

Among 122 significant canonical pathways identified by IPA (*p*-value < 0.05), only four pathways had absolute *z*-scores more than 2.0 and were marked as significantly inhibited in the ICM group (Figure 3). The DEGs involved in these pathways were summarized in Supplementary Table S2. Among these DEGs, *ATP1A1* was involved in the Superpathway of Inositol Phosphate Compounds and also contributed to both heart failure and arrhythmia (Supplementary Table S2, Figure 2C). *ACTC1* and *TGFBR2* contributing to arrhythmia were also involved in EIF2 Signaling Pathway and Senescence Pathway, respectively (Figure 2A, Supplementary Table S2). *MTOR*, *EP300* and *PPP3CC* in the Senescence Pathway were also involved in failure of heart (Figure 2B, Supplementary Table S2).

### 2.5. Upstream Regulator Analysis

**Figure 4:**
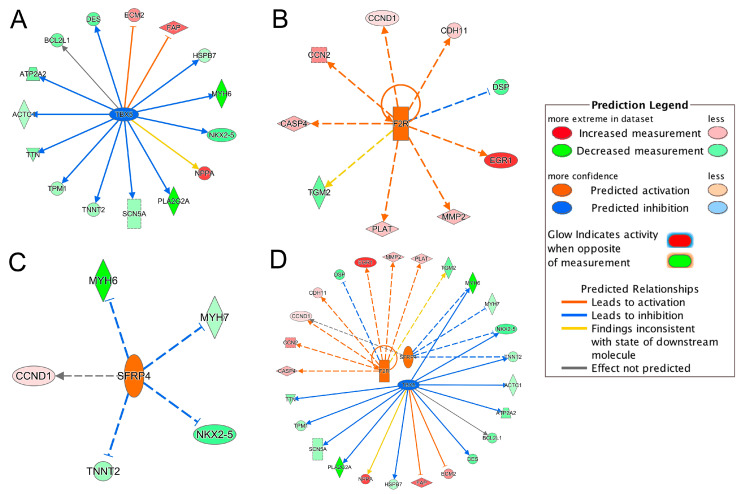
Inhibited/activated upstream regulators and their targeted genes. (**A**) *TBX5*; (**B**) *F2R*; (**C**) *SFRP4*; (**D**) An integrated network of *TBX5*, *F2R* and *SFRP4*. All the targeted genes were differentially expressed based on the meta-analysis. For example, the activation of *F2R* leads to the overexpression (indicated by the orange arrow line) of *CASP4* (indicated by the red color). For other indicators, please refer to the Prediction Legend.

IPA upstream regulator analysis identified 61 significant upstream regulators (*p*-value < 0.05) that were 38 downregulated and 23 upregulated in the ICM group, respectively (Supplementary Table S3). Among those upstream regulators, only *TBX5* was marked as a significantly inhibited regulator (*z*-score = −2.89); only *F2R* and *SFRP4* were significantly activated regulators (*z*-scores = 2.56 and 2.00, respectively) (Supplementary Table S3). Figure 4 summarized the targeted genes by these three upstream regulators.

**Figure 5:**
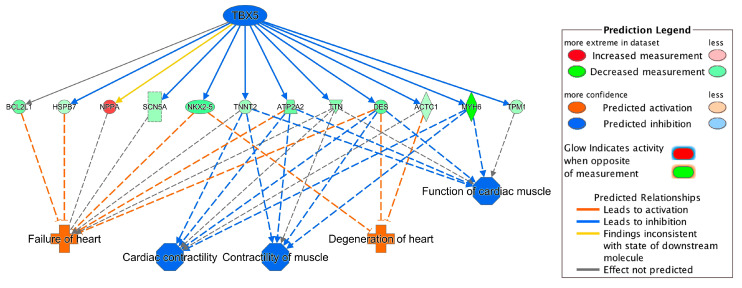
Network of *TBX5*, its targeted genes and corresponding cardiac disorders. For example, inhibition of *TBX5* leads to downregulation (indicated by blue line) of *DES* (shown by green color). Downregulation of *DES* further activates failure of heart (indicated by orange color). For other indicators, please refer to the Prediction Legend.

Integrating the results of the *TBX5*-targeted genes and the DEGs in toxicity pathways, we found that the dysregulation of *TBX5*-targeted genes, *TNNT2, NPPA, TTN, ATP2A2, DES* and *SCN5A*, contributed to the development of heart failure and arrhythmia (Figure 2C and Figure 4A). *NKX2-5*, *HSPB7* and *BCL2L1* were also involved in failure of heart (Figure 2B and Figure 4A). *TBX5*-targeted genes, *MYH6*, *ACTC1* and *TPM1*, were involved in arrhythmia (Figure 2A and Figure 4A). Moreover, *ACTC1* in EIF2 Signaling Pathway was also regulated by *TBX5*. Among *TBX5*-targeted genes, *MYH6*, *TNNT2*, *ECM2*, *TPM1* and *DES*, were found in the top 50 DEGs list (Table 2). The network of *TBX5*, its targeted genes and the corresponding cardiac disorders via IPA regulator effects analysis further indicated an important role of *TBX5* in the development of HF-related dysfunctions and diseases (Figure 5). Inhibited *TBX5* caused dysregulation of several genes such as *BCL2L1*, *HSPB7*, *NPPA, SCN5A*, *NKX2-5*, *TNNT2*, *ATP2A2*, *TTN* and *DES*, which are involved in the activation of heart failure (Figure 5). Inhibited *TBX5* also contributed to other cardiac dysfunctions including degeneration of heart (increased), cardiac contractility (decreased), contractility of muscle (decreased) and function of cardiac muscle (decreased) (Figure 5).

*F2R*, as the activated upstream regulator, upregulated *PLAT*, which was involved in heart failure and arrhythmia (Figure 2C and Figure 4B). Increased *CCN2*, regulated by *F2R*, contributed to failure of heart (Figure 2B and Figure 4B). Dysregulated *MMP2*, *DSP* and *TGM2* were involved in arrhythmia (Figure 2A and Figure 4B). *SFRP4*, as the other activated upstream regulator, inhibited *TNNT2* and *MYH7*, which contributed to the development of heart failure and arrhythmia (Figure 2C and Figure 4C). Upregulation of *SFRP4* also inhibited the expression of *NKX2-5* and *MYH6* (Figure 4C). *NKX2-5* and *MYH6* were involved in failure of heart and arrhythmia, respectively (Figure 2).

Interestingly, an integrated network of the three upstream regulators and their targeted genes showed that *MYH6*, *NKX2-5* and *TNNT2* were regulated by both *TBX5* and *SFRP4* (Figure 4D). *CCND1* was also regulated by both *SFRP4* and *F2R* (Figure 4D). As mentioned above, some of these regulated genes were involved in failure of heart and/or arrhythmia (Figure 2C), indicating that the three key upstream regulators are common hubs regulating the downstream genes importantly contributing to HF-related cardiac disorders.

## 3. Discussion

In this study, we performed the first RNA-seq meta-analysis in the field of clinical ICM using four RNA-seq studies to profile gene expression signatures and identify key transcriptional regulators. We applied a consistent bioinformatics pipeline for processing the raw RNA-seq data (FASTQ files) of all four individual datasets to prevent methodological inconsistences in terms of data processing and bioinformatics pipelines among original studies. Our meta-analysis identified a total of 911 differentially expressed genes including 582 downregulated and 329 upregulated genes (Supplementary Table S1).

Among the top 50 significant DEGs (Table 2), several genes, such as *OGN* and *RPL26*, were previously reported to be dysregulated in ICM patients [15,16]. Upregulation of *OGN*, osteoglycin, has been reported to play a role in collagen maturation and deposition in mouse myocardial infarction tissue [15]. Increased circulating *OGN* has also been observed in ischemic HF patients experiencing myocardial infarction compared to patients with non-ischemic HF and thus it has been proposed as a biomarker for ischemic HF with pre-existing myocardial infarction [15]. Moreover, upregulation of *RPL26*, ribosomal protein L26, has also been observed in patient with ischemic HF [16]. Abnormal expressions of *DES* and *PTN* were also reported in dilated cardiomyopathy (DCM) [17,18]. Interestingly, genetic variants in several genes of the top 50 DEGs were reported to be associated with ICM and other heart diseases. For example, genetic variants of *PALLD* (palladin, cytoskeletal associated protein), important for organizing actin cytoskeleton, have been reported to be associated with myocardial infarction [19,20]. Genetic variants of *MYH6*, *TNNT2* and *TPM1*, have been found to be associated with hypoplastic left heart, cardiac hypertrophy and DCM, respectively [21,22,23].

Meta-analysis is a more sensitive and reliable approach to identify novel robust DEGs due to its greater power to detect differential expression [9]. Fifty-four new DEGs were discovered through our meta-analysis and they were not detected by analyzing the individual datasets. Most of these genes have not been reported prior as related to ICM. However, some of these DEGs have been found to be associated with HF-related diseases. For example, *CRMP1* has been demonstrated as a potential candidate for left-sided congenital heart disease [24]. Lower expression of *ATP1A1* has been reported in end-stage HF [25]. Consistently, our IPA pathway analysis showed that *ATP1A1* was involved in heart failure (Figure 2B). Abnormal expressions of *AZGP1* in chronic HF [26] and *MDFIC* in DCM have been previously reported [27]. Moreover, genetic mutation in *PKD2* has been reported in idiopathic DCM [28]. Further research is needed to investigate pathophysiological mechanisms of these newly identified genes in our meta-analysis.

*ACE2*, *SP100*, *CITED2*, *CEBPD*, *BCL3*, *CREB*, *SMARCA4*, *NCAM1* and *SFRP4* have been previously reported as transcriptional regulators in heart failure [29,30,31,32,33,34]. Although *CITED2*, *CREB5* (belongs to CREB family), *CREB3L1* (belongs to CREB family), *SMARCA4*, *NCAM1* and *SFRP4* were significant DEGs in our dataset (Supplementary Table S1), only *SFRP4* was identified as the significantly activated transcriptional regulator based on significant activation *z*-score (absolute *z*-score > 2.0) from our IPA analysis (Figure 4). Upstream regulator analysis also identified two additional transcriptional regulators, *TBX5* and *F2R* (Figure 4). *TBX5*, a member of the T-box transcription factor family, was the top inhibited regulator. It has been previously reported that malfunction of *TBX5* could lead to several cardiovascular diseases during embryonic development and also during adulthood [35]. In our study, inhibition of *TBX5* was shown to dysregulate several genes such as *DES*, *NKX2-5*, *ACTC1*, *MYH6*, *ATP2A2* and *HSPB7*, which further contributed to ICM-related diseases such as failure of heart and degeneration of heart (Figure 5). Cardiac muscle functions including cardiac contractility, contractility of muscle and function of cardiac muscles were also shown to be influenced due to inhibition of *TBX5* (Figure 5). Consistent with our finding, *TBX5* along with *MEF2C* has been reported to activate the expression of *MYH6*, which is considered as the building block of cardiomyocytes and plays a crucial role in heart development and function [36]. Moreover, *DES* expression has also been reported to be regulated by *TBX5* [37] and a decreased number of *DES*-positive myocytes has been found in ischemic heart failure and was associated with reduced cardiac function [38]. Our study also found that dysregulated *TBX5* could inhibit the expression of *ATP2A2* (Figure 5), an ATPase enzyme that plays an important role in muscle contraction and relaxation, and decreased *ATP2A2* has also been observed during human end-stage heart failure [39,40]. *TBX5*-regulated *NKX2-5* is involved in heart formation and development and dysregulated *NKX2-5* could lead to heart failure and sudden cardiac death [37,41]. Therefore, our results demonstrate a strong association of *TBX5* with heart diseases and further propose its important transcriptional regulatory role in the development of ICM for future mechanistic studies.

*F2R* and *SFRP4* were identified as significantly activated upstream regulators mediating multiple HF-related genes (Figure 2 and Figure 4). *SFRP4*, secreted frizzled-related protein 4, is a member of the *SFRPs* family, functioning as soluble modulators in Wnt signaling [20]. Increased *SFRP4* has been reported in patients with coronary heart disease and DCM [34,42]. Several *SFRP4*-targeted genes, such as *MYH6*, *MYH7*, *TNNT2* and *NKX2-5*, contributed to different types of heart diseases [22,41,43,44]. *F2R*, coagulation factor II receptor, is a member of the G-protein coupled receptor family and it is important for regulating the thrombotic response [20]. Genetics variants in *F2R* have been reported to influence the risk of myocardial infarction and coronary heart disease [45,46]. In our study, activation of *F2R* was indicated to cause overexpression of several genes, including *CCN2*, *CCND1*, *CDH11*, *CASP4*, *EGR1*, *MMP2* and *PLAT*, in the ICM group (Figure 4B). Activation of *SFRP4* and *F2R* also upregulated *CCND1* (Figure 4D), which was involved in EIF2 Signaling and Senescence Pathways (Supplementary Table S2). *CCND1* (Cyclin D1) has been reported to promote cardiomyocyte division in vivo and regulate cardiac function responding to heart failure in a rat myocardial infarction model [47]. Besides *TBX5*, our study identified *F2R* and *SFRP4* as two important activated transcriptional regulators involved in the development of ICM-related cardiac dysfunctions.

In conclusion, our study, which is the first RNA-seq meta-analysis in the field of clinical ICM, identified multiple novel dysregulated genes and three key transcriptional regulators involved in the development of ischemic cardiomyopathy and its associated cardiovascular diseases. The three transcriptional regulators could be further examined as potential biomarkers for simultaneous regulation of multiple ICM-involved genes to develop novel diagnostic and therapeutic strategies in ischemic heart failure.

## 4. Materials and Methods

**Figure 6:**
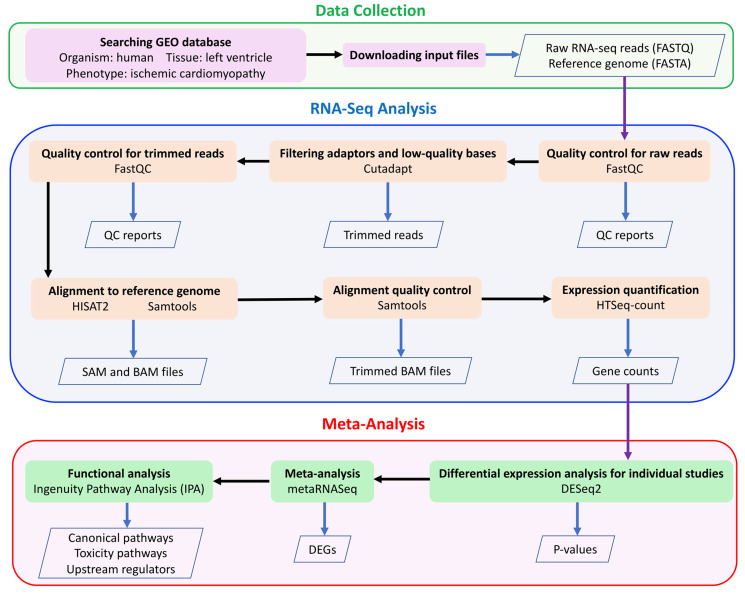
The meta-analysis workflow.

Table 1 summarizes four RNA-seq studies of ICM using tissue samples from human left ventricle, found in NCBI GEO [48] database (https://www.ncbi.nlm.nih.gov/geo/). Further, we did not include the datasets if they were not collected using the Illumina sequencing platform or they were collected in patients with any treatment of a specific drug or medically implanted device. The clinical information of ICM patients and their controls has been described in Study_1 [49], Study_2 [50], Study_3 [51] and Study_4 [14]. RNA-seq analyses of these four individual studies were previously published [49,50,52,53], thus all four individual datasets have been validated for our current meta-analysis. Detailed information of the data processing and bioinformatics analysis has been described in our recently published paper [32] and is shown in Figure 6. Briefly, FASTQ files were downloaded from the European Nucleotide Archive website (https://www.ebi.ac.uk/ena). Quality control for raw reads and trimmed reads was performed using FastQC [54]. Adaptors and low-quality bases (Phred quality score < 10) were filtered using Cutadapt [55]. A pipeline of HISAT2 [56], Samtools [57] and HTSeq-count [58] was used for aligning the trimmed reads to the human reference genome (GRCh38) and quantifying gene expression. Only uniquely mapped reads were used for expression quantification.

DESeq2 [59] was used to perform differential expression analysis. Genes with low read counts were filtered with default parameters in DESeq2. Quantitative meta-analysis was performed through Fisher’s combined probability test [60] using metaRNASeq [9]. Raw *p*-values were adjusted by the Benjamini–Hochberg false discovery rate (FDR) method and the adjusted *p*-values less than 0.05 were considered as statistically significant. Only DEGs with consistent expression directions among the four individual studies were included in the final DEGs list.

To identify enriched canonical pathways, toxicity functions (IPA-Tox) and upstream transcriptional regulators, the Ingenuity Pathway Analysis software (IPA, Qiagen, Redwood City, CA, USA) [61] was used to analyze DEGs identified by meta-analysis. VennDiagram [62] in R was used to generate a Venn diagram of common DEGs among the meta-analysis and individual studies. A heatmap of the DEGs identified by meta-analysis was generated using the heatmap.2 function from the gplots package in R [63]. The compute-intensive tasks were performed using Ohio Supercomputer Center [64].

## Figures and Tables

**Table 1:** GEO datasets used for meta-analysis.

Study	Dataset	Platform	Sample Size	Tissue
Study_1	GSE116250 [11]	Illumina HiSeq 2500	13 ICM 14 NF	left ventricle
Study_2	GSE120852 [12]	Illumina HiSeq 2500	5 ICM 5 NF	left ventricle
Study_3	GSE46224 [13]	Illumina HiSeq 2000	8 ICM 8 NF	left ventricle
Study_4	GSE48166 [14]	Illumina Genome Analyzer II	15 ICM 15 NF	left ventricle

**Table 2:** Top 50 DEGs identified in meta-analysis.

Ensembl_ID	Gene_Symbol	Adj_P ^1^	Average_Log_2_FC ^2^	Effect ^3^
ENSG00000008311	*AASS*	0	−1.03	Down
ENSG00000075413	*MARK3*	0	−0.88	Down
ENSG00000076351	*SLC46A1*	0	0.48	Up
ENSG00000100196	*KDELR3*	0	0.72	Up
ENSG00000103415	*HMOX2*	0	−0.81	Down
ENSG00000105894	*PTN*	0	1.47	Up
ENSG00000106809	*OGN*	0	2.26	Up
ENSG00000106819	*ASPN*	0	1.99	Up
ENSG00000106823	*ECM2*	0	1.17	Up
ENSG00000118194	*TNNT2*	0	−0.55	Down
ENSG00000122034	*GTF3A*	0	−0.54	Down
ENSG00000123689	*G0S2*	0	−1.57	Down
ENSG00000126106	*TMEM53*	0	−0.58	Down
ENSG00000129250	*KIF1C*	0	−0.56	Down
ENSG00000130528	*HRC*	0	−0.58	Down
ENSG00000139329	*LUM*	0	1.81	Up
ENSG00000140416	*TPM1*	0	−0.53	Down
ENSG00000141905	*NFIC*	0	−0.57	Down
ENSG00000145934	*TENM2*	0	−0.67	Down
ENSG00000156219	*ART3*	0	−1.31	Down
ENSG00000161970	*RPL26*	0	−0.76	Down
ENSG00000175084	*DES*	0	−0.80	Down
ENSG00000176293	*ZNF135*	0	0.51	Up
ENSG00000179526	*SHARPIN*	0	−0.34	Down
ENSG00000197256	*KANK2*	0	−0.57	Down
ENSG00000197616	*MYH6*	0	−2.59	Down
ENSG00000198542	*ITGBL1*	0	1.69	Up
ENSG00000198624	*CCDC69*	0	−0.87	Down
ENSG00000210127	*MT-TA*	0	−1.66	Down
ENSG00000233098	*CCDC144NL-AS1*	0	1.17	Up
ENSG00000140319	*SRP14*	6.91 ×10^−14^	−0.53	Down
ENSG00000197586	*ENTPD6*	6.91 × 10^−14^	−0.77	Down
ENSG00000267280	*TBX2-AS1*	6.91 × 10^−14^	0.83	Up
ENSG00000152086	*TUBA3E*	1.33 × 10^−13^	−1.99	Down
ENSG00000170448	*NFXL1*	1.33 × 10^−13^	−1.84	Down
ENSG00000165124	*SVEP1*	1.89 × 10^−13^	1.24	Up
ENSG00000152580	*IGSF10*	2.46 × 10^−13^	1.54	Up
ENSG00000143603	*KCNN3*	3.04 × 10^−13^	1.43	Up
ENSG00000187837	*HIST1H1C*	3.60 × 10^−13^	−0.89	Down
ENSG00000075886	*TUBA3D*	4.15 × 10^−13^	−1.60	Down
ENSG00000189060	*H1F0*	8.87 × 10^−13^	−0.80	Down
ENSG00000134247	*PTGFRN*	2.21 × 10^−12^	0.93	Up
ENSG00000116690	*PRG4*	3.94 × 10^−12^	0.84	Up
ENSG00000160392	*C19orf47*	4.61 × 10^−12^	−0.95	Down
ENSG00000129009	*ISLR*	7.14 × 10^−12^	1.49	Up
ENSG00000129116	*PALLD*	8.77 × 10^−12^	−0.72	Down
ENSG00000173991	*TCAP*	1.11 × 10^−11^	−0.55	Down
ENSG00000104879	*CKM*	1.27 × 10^−11^	−0.79	Down
ENSG00000108298	*RPL19*	1.66 × 10^−11^	−0.54	Down
ENSG00000142748	*FCN3*	1.75 × 10^−11^	−1.59	Down

^1^: FDR-adjusted *p*-value, Adj_P = 0 indicates that the FDR-adjusted *p*-value was very small and rounded down to 0; ^2^: Average of log_2_FC from individual studies, FC: fold-change; ^3^: “Up” or “Down” indicates whether the gene was upregulated or downregulated.

**Table 3:** DEGs identified only by meta-analysis.

Ensembl_ID	Gene_Symbol	Adj_P ^1^	Average_Log_2_FC ^2^	Effect ^3^
ENSG00000171517	*LPAR3*	8.64 × 10^−4^	−1.16	Down
ENSG00000178607	*ERN1*	2.24× 10^−3^	0.88	Up
ENSG00000048707	*VPS13D*	3.17 × 10^−3^	−0.60	Down
ENSG00000179604	*CDC42EP4*	3.18 × 10^−3^	−0.66	Down
ENSG00000072832	*CRMP1*	3.42 × 10^−3^	0.75	Up
ENSG00000162458	*FBLIM1*	6.49 × 10^−3^	−0.79	Down
ENSG00000178307	*TMEM11*	6.86 × 10^−3^	−0.49	Down
ENSG00000166278	*C2*	7.49 × 10^−3^	1.02	Up
ENSG00000228526	*MIR34AHG*	8.17 × 10^−3^	0.97	Up
ENSG00000184007	*PTP4A2*	8.29 × 10^−3^	−0.40	Down
ENSG00000160818	*GPATCH4*	8.45 × 10^−3^	−0.48	Down
ENSG00000100949	*RABGGTA*	8.63 × 10^−3^	−0.38	Down
ENSG00000255248	*MIR100HG*	9.29 × 10^−3^	0.35	Up
ENSG00000165028	*NIPSNAP3B*	9.48 × 10^−3^	−0.52	Down
ENSG00000133678	*TMEM254*	9.98 × 10^−3^	0.63	Up
ENSG00000128272	*ATF4*	1.02 × 10^−2^	−0.55	Down
ENSG00000103342	*GSPT1*	1.13 × 10^−2^	−0.33	Down
ENSG00000163866	*SMIM12*	1.27 × 10^−2^	−0.46	Down
ENSG00000198355	*PIM3*	1.29 × 10^−2^	−0.61	Down
ENSG00000163399	*ATP1A1*	1.32 × 10^−2^	−0.47	Down
ENSG00000160862	*AZGP1*	1.33 × 10^−2^	−0.85	Down
ENSG00000180758	*GPR157*	1.42 × 10^−2^	−0.73	Down
ENSG00000115461	*IGFBP5*	1.53 × 10^−2^	0.48	Up
ENSG00000037280	*FLT4*	1.76 × 10^−2^	0.48	Up
ENSG00000135272	*MDFIC*	1.78 × 10^−2^	0.69	Up
ENSG00000131781	*FMO5*	1.83 × 10^−2^	−0.73	Down
ENSG00000184887	*BTBD6*	1.93 × 10^−2^	−0.53	Down
ENSG00000142494	*SLC47A1*	2.39 × 10^−2^	0.58	Up
ENSG00000113811	*SELENOK*	2.63 × 10^−2^	−0.34	Down
ENSG00000186567	*CEACAM19*	2.63 × 10^−2^	−0.60	Down
ENSG00000100767	*PAPLN*	2.66 × 10^−2^	0.97	Up
ENSG00000159674	*SPON2*	2.85 × 10^−2^	0.62	Up
ENSG00000169155	*ZBTB43*	2.95 × 10^−2^	−0.40	Down
ENSG00000103034	*NDRG4*	3.09 × 10^−2^	−0.34	Down
ENSG00000106034	*CPED1*	3.11 × 10^−2^	0.37	Up
ENSG00000179262	*RAD23A*	3.28 × 10^−2^	−0.32	Down
ENSG00000169718	*DUS1L*	3.31 × 10^−2^	−0.37	Down
ENSG00000107736	*CDH23*	3.35 × 10^−2^	0.71	Up
ENSG00000108883	*EFTUD2*	3.43 × 10^−2^	−0.26	Down
ENSG00000139990	*DCAF5*	3.45 × 10^−2^	−0.34	Down
ENSG00000019995	*ZRANB1*	3.49 × 10^−2^	−0.24	Down
ENSG00000160877	*NACC1*	3.49 × 10^−2^	−0.50	Down
ENSG00000175602	*CCDC85B*	3.51 × 10^−2^	0.56	Up
ENSG00000143869	*GDF7*	3.65 × 10^−2^	0.76	Up
ENSG00000182287	*AP1S2*	3.90 × 10^−2^	−0.34	Down
ENSG00000114670	*NEK11*	3.94 × 10^−2^	0.77	Up
ENSG00000197977	*ELOVL2*	3.96 × 10^−2^	−0.71	Down
ENSG00000170004	*CHD3*	4.20 × 10^−2^	0.48	Up
ENSG00000168615	*ADAM9*	4.38 × 10^−2^	−0.39	Down
ENSG00000118762	*PKD2*	4.42 × 10^−2^	0.32	Up
ENSG00000078061	*ARAF*	4.54 × 10^−2^	−0.27	Down
ENSG00000113140	*SPARC*	4.57 × 10^−2^	0.49	Up
ENSG00000144645	*OSBPL10*	4.62 × 10^−2^	0.85	Up
ENSG00000003096	*KLHL13*	4.99 × 10^−2^	0.83	Up

^1^: FDR-adjusted *p*-value; ^2^: Average of log_2_FC from individual studies, FC: fold-change; ^3^: “Up” or “Down” indicates whether the gene was upregulated or downregulated.

**Table 4:** Shared DEGs between arrhythmia and failure of heart.

Ensembl_ID	Gene_Symbol	Average_Log_2_FC ^1^	Effect ^2^
ENSG00000120907	*ADRA1A*	−0.67	Down
ENSG00000163399	*ATP1A1*	−0.47	Down
ENSG00000174437	*ATP2A2*	−0.74	Down
ENSG00000007402	*CACNA2D2*	0.59	UP
ENSG00000175084	*DES*	−0.80	Down
ENSG00000126218	*F10*	0.54	Up
ENSG00000152642	*GPD1L*	−0.75	Down
ENSG00000171385	*KCND3*	−0.91	Down
ENSG00000055118	*KCNH2*	−0.84	Down
ENSG00000187486	*KCNJ11*	−1.03	Down
ENSG00000134571	*MYBPC3*	−0.55	Down
ENSG00000092054	*MYH7*	−0.45	Down
ENSG00000175206	*NPPA*	1.86	UP
ENSG00000104368	*PLAT*	0.64	Up
ENSG00000183873	*SCN5A*	−0.54	Down
ENSG00000118194	*TNNT2*	−0.55	Down
ENSG00000155657	*TTN*	−0.66	Down

^1^: Average of log_2_FC from individual studies, FC: fold-change; ^2^: “Up” or “Down” indicates whether the gene was upregulated or downregulated.

## Supplementary Materials

Supplementary materials can be found at https://www.mdpi.com/1422-0067/21/10/3472/s1, Table S1: A complete list of differential expressed genes identified in meta-analysis; Table S2: Significant canonical pathways with absolute *z*-score > 2.0 and their involved genes; Table S3: Differentially expressed upstream regulators and their targeted genes.
