# Supplementary material for: Identification of Upstream Transcriptional Regulators of Ischemic Cardiomyopathy Using Cardiac RNA-Seq Meta-Analysis
Source: Int J Mol Sci. 2020 May 14;21(10):3472. doi: 10.3390/ijms21103472 (PMC7278960; doi:10.3390/ijms21103472)
Supplement: Supplementary file 1 [file ijms-21-03472-s001.pdf]

Supplementary Tables

Supplementary Table S1. A complete list of differential expressed genes identified in meta-analysis.

Ensembl_ID	Gene_Symbol	DE_1[1]	DE_2[1]	DE_3[1]	DE_4[1]	Adj_P[2]	Average_Log_2FC[3]	Effect[4]
ENSG00000008311	*AASS*	1	1	1	0	0.00E+00	-1.03	Down
ENSG00000075413	*MARK3*	1	1	1	0	0.00E+00	-0.88	Down
ENSG00000076351	*SLC46A1*	1	0	1	0	0.00E+00	0.48	Up
ENSG00000100196	*KDELR3*	1	0	1	0	0.00E+00	0.72	Up
ENSG00000103415	*HMOX2*	1	1	1	0	0.00E+00	-0.81	Down
ENSG00000105894	*PTN*	0	1	1	0	0.00E+00	1.47	Up
ENSG00000106809	*OGN*	1	1	1	0	0.00E+00	2.26	Up
ENSG00000106819	*ASPN*	1	1	1	0	0.00E+00	1.99	Up
ENSG00000106823	*ECM2*	0	1	1	0	0.00E+00	1.17	Up
ENSG00000118194	*TNNT2*	1	0	1	0	0.00E+00	-0.55	Down
ENSG00000122034	*GTF3A*	1	0	1	0	0.00E+00	-0.54	Down
ENSG00000123689	*G0S2*	1	0	0	0	0.00E+00	-1.57	Down
ENSG00000126106	*TMEM53*	1	1	0	0	0.00E+00	-0.58	Down
ENSG00000129250	*KIF1C*	1	0	0	0	0.00E+00	-0.56	Down
ENSG00000130528	*HRC*	1	0	0	0	0.00E+00	-0.58	Down
ENSG00000139329	*LUM*	1	1	1	0	0.00E+00	1.81	Up
ENSG00000140416	*TPM1*	1	0	0	0	0.00E+00	-0.53	Down
ENSG00000141905	*NFIC*	1	1	0	0	0.00E+00	-0.57	Down
ENSG00000145934	*TENM2*	1	0	0	0	0.00E+00	-0.67	Down
ENSG00000156219	*ART3*	1	0	1	0	0.00E+00	-1.31	Down
ENSG00000161970	*RPL26*	1	0	0	0	0.00E+00	-0.76	Down
ENSG00000175084	*DES*	1	1	0	0	0.00E+00	-0.80	Down
ENSG00000176293	*ZNF135*	1	0	0	0	0.00E+00	0.51	Up
ENSG00000179526	*SHARPIN*	1	0	0	0	0.00E+00	-0.34	Down
ENSG00000197256	*KANK2*	1	0	0	0	0.00E+00	-0.57	Down
ENSG00000197616	*MYH6*	1	0	1	1	0.00E+00	-2.59	Down
ENSG00000198542	*ITGBL1*	1	1	1	0	0.00E+00	1.69	Up
ENSG00000198624	*CCDC69*	1	0	1	0	0.00E+00	-0.87	Down
ENSG00000210127	*MT-TA*	1	0	1	0	0.00E+00	-1.66	Down
ENSG00000233098	*CCDC144NL-AS1*	1	1	1	0	0.00E+00	1.17	Up
ENSG00000140319	*SRP14*	1	0	0	0	6.91E-14	-0.53	Down
ENSG00000197586	*ENTPD6*	1	0	1	0	6.91E-14	-0.77	Down
ENSG00000267280	*TBX2-AS1*	1	0	0	0	6.91E-14	0.83	Up
ENSG00000152086	*TUBA3E*	1	1	1	0	1.33E-13	-1.99	Down
ENSG00000170448	*NFXL1*	1	0	1	0	1.33E-13	-1.84	Down
ENSG00000165124	*SVEP1*	1	1	1	0	1.89E-13	1.24	Up
ENSG00000152580	*IGSF10*	1	1	0	0	2.46E-13	1.54	Up
ENSG00000143603	*KCNN3*	0	1	1	0	3.04E-13	1.43	Up
ENSG00000187837	*HIST1H1C*	1	0	0	0	3.60E-13	-0.89	Down
ENSG00000075886	*TUBA3D*	0	1	1	0	4.15E-13	-1.60	Down
ENSG00000189060	*H1F0*	1	1	0	0	8.87E-13	-0.80	Down
ENSG00000134247	*PTGFRN*	1	0	1	0	2.21E-12	0.93	Up
ENSG00000116690	*PRG4*	1	0	1	0	3.94E-12	0.84	Up
ENSG00000160392	*C19orf47*	1	1	1	0	4.61E-12	-0.95	Down
ENSG00000129009	*ISLR*	0	1	1	0	7.14E-12	1.49	Up
ENSG00000129116	*PALLD*	0	1	1	0	8.77E-12	-0.72	Down
ENSG00000173991	*TCAP*	1	0	0	0	1.11E-11	-0.55	Down

ENSG00000104879	*CKM*	1	0	1	0	1.27E-11	-0.79	Down
ENSG00000108298	*RPL19*	1	0	0	0	1.66E-11	-0.54	Down
ENSG00000142748	*FCN3*	0	1	1	0	1.75E-11	-1.59	Down
ENSG00000211455	*STK38L*	1	1	1	0	2.14E-11	1.16	Up
ENSG00000197860	*SGTB*	1	0	0	0	2.50E-11	0.77	Up
ENSG00000167646	*DNAAF3*	1	0	0	0	4.36E-11	1.34	Up
ENSG00000187955	*COL14A1*	0	1	1	0	4.92E-11	1.99	Up
ENSG00000131174	*COX7B*	1	0	1	0	6.04E-11	-1.09	Down
ENSG00000143196	*DPT*	0	1	1	0	6.23E-11	1.36	Up
ENSG00000210107	*MT-TQ*	1	0	0	0	7.87E-11	-1.08	Down
ENSG00000149294	*NCAM1*	1	1	0	0	1.00E-10	-0.71	Down
ENSG00000136942	*RPL35*	1	0	0	0	1.08E-10	-0.68	Down
ENSG00000138326	*RPS24*	1	0	0	0	1.75E-10	-0.75	Down
ENSG00000115593	*SMYD1*	0	0	1	0	1.82E-10	-0.64	Down
ENSG00000177463	*NR2C2*	1	0	1	0	3.54E-10	-0.41	Down
ENSG00000113296	*THBS4*	1	0	1	0	3.85E-10	1.42	Up
ENSG00000114867	*EIF4G1*	1	0	0	0	3.85E-10	-0.66	Down
ENSG00000138074	*SLC5A6*	1	0	0	0	4.23E-10	-0.40	Down
ENSG00000175416	*CLTB*	1	0	0	0	4.49E-10	-0.69	Down
ENSG00000106624	*AEBP1*	1	0	1	0	4.52E-10	1.69	Up
ENSG00000136436	*CALCOCO2*	1	1	1	0	5.08E-10	-0.76	Down
ENSG00000110400	*NECTIN1*	1	1	1	0	5.36E-10	-1.60	Down
ENSG00000213694	*S1PR3*	1	0	1	0	5.40E-10	-1.12	Down
ENSG00000096696	*DSP*	1	0	0	0	5.87E-10	-0.84	Down
ENSG00000114923	*SLC4A3*	1	0	0	0	6.17E-10	-0.80	Down
ENSG00000174437	*ATP2A2*	1	0	1	0	6.19E-10	-0.74	Down
ENSG00000188229	*TUBB4B*	1	0	1	0	8.15E-10	-0.82	Down
ENSG00000065054	*SLC9A3R2*	1	0	1	0	8.20E-10	-0.65	Down
ENSG00000118564	*FBXL5*	0	0	1	0	9.08E-10	-0.52	Down
ENSG00000182979	*MTA1*	1	0	0	0	9.82E-10	-0.79	Down
ENSG00000171533	*MAP6*	1	0	1	0	1.17E-09	0.65	Up
ENSG00000147872	*PLIN2*	1	1	1	0	1.18E-09	-1.38	Down
ENSG00000179954	*SSC5D*	0	1	1	0	1.18E-09	1.54	Up
ENSG00000184441	*AP001062.1*	1	0	0	0	1.24E-09	0.33	Up
ENSG00000147687	*TATDN1*	1	0	0	0	1.39E-09	-0.49	Down
ENSG00000148187	*MRRF*	1	0	1	0	1.61E-09	-0.54	Down
ENSG00000178685	*PARP10*	1	1	1	0	1.65E-09	0.70	Up
ENSG00000196535	*MYO18A*	1	0	0	0	1.72E-09	-0.47	Down
ENSG00000142910	*TINAGL1*	1	0	1	0	1.80E-09	-0.64	Down
ENSG00000134571	*MYBPC3*	1	0	0	0	1.93E-09	-0.55	Down
ENSG00000159166	*LAD1*	1	1	0	0	1.93E-09	-1.95	Down
ENSG00000144857	*BOC*	0	1	1	0	1.97E-09	0.98	Up
ENSG00000109099	*PMP22*	1	0	0	0	2.60E-09	-0.54	Down
ENSG00000173402	*DAG1*	1	1	0	0	2.86E-09	-0.65	Down
ENSG00000135720	*DYNC1LI2*	1	0	1	0	3.77E-09	0.60	Up
ENSG00000087074	*PPP1R15A*	1	0	0	0	3.83E-09	0.72	Up
ENSG00000135919	*SERPINE2*	0	0	1	0	4.36E-09	0.90	Up
ENSG00000054654	*SYNE2*	1	0	1	0	5.27E-09	-0.80	Down
ENSG00000157613	*CREB3L1*	1	0	1	0	5.41E-09	-0.79	Down
ENSG00000106483	*SFRP4*	0	0	1	0	6.21E-09	2.62	Up
ENSG00000254473	*AL354920.1*	1	0	0	0	6.29E-09	0.43	Up
ENSG00000248527	*MTATP6P1*	1	1	0	0	7.06E-09	-0.75	Down
ENSG00000198763	*MT-ND2*	1	0	0	0	8.19E-09	-0.72	Down

ENSG00000239474	*KLHL41*	0	1	1	0	8.19E-09	-0.89	Down
ENSG00000120833	*SOCS2*	1	0	1	0	1.01E-08	0.90	Up
ENSG00000078098	*FAP*	0	0	1	0	1.03E-08	1.57	Up
ENSG00000164530	*PI16*	0	1	1	0	1.33E-08	1.65	Up
ENSG00000185033	*SEMA4B*	0	1	0	0	1.37E-08	-0.82	Down
ENSG00000090006	*LTBP4*	0	1	0	0	1.42E-08	0.80	Up
ENSG00000182107	*TMEM30B*	0	1	1	0	1.51E-08	1.88	Up
ENSG00000065978	*YBX1*	1	0	1	0	1.59E-08	-0.57	Down
ENSG00000011028	*AC080038.1*	1	0	1	0	1.61E-08	1.26	Up
ENSG00000162576	*MXRA8*	1	1	1	0	1.84E-08	0.91	Up
ENSG00000149575	*SCN2B*	0	1	1	0	2.07E-08	1.04	Up
ENSG00000174348	*PODN*	0	1	0	0	2.11E-08	1.13	Up
ENSG00000171385	*KCND3*	1	0	1	0	2.40E-08	-0.91	Down
ENSG00000107404	*DVL1*	1	0	1	0	2.69E-08	-0.68	Down
ENSG00000198888	*MT-ND1*	1	0	1	0	2.72E-08	-0.71	Down
ENSG00000186187	*ZNRF1*	1	0	0	0	2.85E-08	-0.33	Down
ENSG00000026950	*BTN3A1*	0	1	0	0	2.88E-08	0.91	Up
ENSG00000113369	*ARRDC3*	1	1	0	0	2.99E-08	0.96	Up
ENSG00000101825	*MXRA5*	0	0	1	0	3.58E-08	1.59	Up
ENSG00000101187	*SLCO4A1*	0	0	1	0	4.18E-08	-1.21	Down
ENSG00000120729	*MYOT*	1	1	0	0	4.41E-08	-1.30	Down
ENSG00000166482	*MFAP4*	1	1	1	0	4.90E-08	1.29	Up
ENSG00000150776	*NKAPD1*	1	0	0	0	5.47E-08	-0.47	Down
ENSG00000119707	*RBM25*	1	0	0	0	5.63E-08	-0.38	Down
ENSG00000167522	*ANKRD11*	1	1	0	0	5.99E-08	-0.45	Down
ENSG00000063245	*EPN1*	1	1	1	0	6.23E-08	-0.79	Down
ENSG00000158747	*NBL1*	1	1	1	0	6.32E-08	1.06	Up
ENSG00000204569	*PPP1R10*	0	0	1	0	6.88E-08	0.60	Up
ENSG00000155657	*TTN*	1	1	1	0	7.45E-08	-0.66	Down
ENSG00000198517	*MAFK*	1	0	1	0	7.78E-08	0.72	Up
ENSG00000210117	*MT-TW*	1	0	1	0	9.17E-08	-1.62	Down
ENSG00000255690	*TRIL*	0	1	1	0	9.35E-08	1.35	Up
ENSG00000160469	*BRSK1*	0	0	1	0	1.06E-07	0.95	Up
ENSG00000182197	*EXT1*	1	0	1	0	1.14E-07	1.11	Up
ENSG00000279088	*AC022400.8*	1	0	0	0	1.14E-07	-0.58	Down
ENSG00000166848	*TERF2IP*	1	0	1	0	1.18E-07	0.47	Up
ENSG00000131730	*CKMT2*	1	0	0	0	1.19E-07	-0.62	Down
ENSG00000161940	*BCL6B*	1	1	1	0	1.20E-07	1.30	Up
ENSG00000105401	*CDC37*	1	0	0	0	1.37E-07	-0.43	Down
ENSG00000020181	*ADGRA2*	1	1	1	0	1.43E-07	0.68	Up
ENSG00000135318	*NT5E*	0	1	1	0	1.46E-07	1.05	Up
ENSG00000110042	*DTX4*	1	1	0	0	1.48E-07	1.25	Up
ENSG00000205363	*INSYN1*	1	0	1	0	1.48E-07	-1.08	Down
ENSG00000163050	*COQ8A*	1	0	1	0	1.64E-07	-0.88	Down
ENSG00000169604	*ANTXR1*	0	1	1	0	1.75E-07	0.97	Up
ENSG00000185010	*F8*	1	0	1	0	1.98E-07	-0.71	Down
ENSG00000270504	*AL391422.4*	1	0	1	0	1.98E-07	0.70	Up
ENSG00000119681	*LTBP2*	0	0	1	0	2.01E-07	1.23	Up
ENSG00000165629	*ATP5F1C*	1	0	0	0	2.20E-07	-0.79	Down
ENSG00000065882	*TBC1D1*	0	1	0	0	2.40E-07	-0.39	Down
ENSG00000138303	*ASCC1*	1	0	0	0	2.55E-07	-0.38	Down
ENSG00000164694	*FNDC1*	1	1	1	0	2.56E-07	2.06	Up
ENSG00000227199	*ST7-AS1*	1	0	0	0	2.56E-07	-0.52	Down

ENSG00000183801	*OLFML1*	0	1	0	0	2.69E-07	0.85	Up
ENSG00000116898	*MRPS15*	1	0	0	0	2.72E-07	-0.77	Down
ENSG00000225614	*ZNF469*	1	1	1	0	2.87E-07	1.38	Up
ENSG00000134716	*CYP2J2*	1	0	0	0	3.06E-07	0.96	Up
ENSG00000122378	*PRXL2A*	0	0	1	0	3.10E-07	-0.56	Down
ENSG00000142733	*MAP3K6*	0	1	1	0	3.41E-07	-0.75	Down
ENSG00000214456	*PLIN5*	1	0	1	0	3.55E-07	-1.11	Down
ENSG00000120738	*EGR1*	1	1	1	0	4.00E-07	2.10	Up
ENSG00000101444	*AHCY*	1	0	1	0	4.12E-07	-0.86	Down
ENSG00000091986	*CCDC80*	0	0	1	0	4.46E-07	1.24	Up
ENSG00000055118	*KCNH2*	1	1	1	0	4.53E-07	-0.84	Down
ENSG00000144560	*VGLL4*	0	1	1	0	4.85E-07	0.38	Up
ENSG00000198892	*SHISA4*	1	1	1	0	5.82E-07	-0.83	Down
ENSG00000099139	*PCSK5*	0	1	0	0	6.00E-07	0.73	Up
ENSG00000181104	*F2R*	0	0	1	0	6.00E-07	0.92	Up
ENSG00000113456	*RAD1*	1	1	0	0	6.66E-07	-0.56	Down
ENSG00000196821	*C6orf106*	1	0	1	0	7.17E-07	-0.50	Down
ENSG00000185739	*SRL*	1	1	0	0	7.23E-07	-0.57	Down
ENSG00000211445	*GPX3*	1	1	1	0	7.23E-07	-0.81	Down
ENSG00000172795	*DCP2*	1	0	0	0	7.30E-07	0.69	Up
ENSG00000076555	*ACACB*	1	0	1	0	7.50E-07	-0.87	Down
ENSG00000161647	*MPP3*	1	1	1	0	7.78E-07	-1.23	Down
ENSG00000187486	*KCNJ11*	0	1	1	0	9.00E-07	-1.03	Down
ENSG00000261801	*LOXL1-AS1*	1	0	1	0	9.55E-07	1.00	Up
ENSG00000173706	*HEG1*	1	0	0	0	9.61E-07	0.64	Up
ENSG00000076344	*RGS11*	1	0	1	0	1.05E-06	0.66	Up
ENSG00000044115	*CTNNA1*	1	1	0	0	1.23E-06	-0.57	Down
ENSG00000147573	*TRIM55*	1	0	0	0	1.27E-06	-0.84	Down
ENSG00000131386	*GALNT15*	1	1	0	0	1.30E-06	-1.57	Down
ENSG00000166831	*RBPMS2*	1	1	1	0	1.36E-06	-0.69	Down
ENSG00000177731	*FLII*	1	0	0	0	1.38E-06	-0.60	Down
ENSG00000232973	*CYP1B1-AS1*	1	0	1	0	1.40E-06	1.18	Up
ENSG00000214530	*STARD10*	1	0	1	0	1.44E-06	-0.86	Down
ENSG00000137573	*SULF1*	0	1	1	0	1.61E-06	1.06	Up
ENSG00000084636	*COL16A1*	0	0	1	0	1.65E-06	0.86	Up
ENSG00000169231	*THBS3*	1	0	1	0	1.65E-06	0.96	Up
ENSG00000105664	*COMP*	0	0	1	0	1.68E-06	2.39	Up
ENSG00000162434	*JAK1*	1	1	1	0	1.75E-06	-0.55	Down
ENSG00000133392	*MYH11*	1	0	0	0	1.77E-06	0.56	Up
ENSG00000133477	*FAM83F*	0	1	1	0	1.78E-06	-1.15	Down
ENSG00000071626	*DAZAP1*	1	0	0	0	2.07E-06	-0.71	Down
ENSG00000188846	*RPL14*	1	0	1	0	2.10E-06	-0.56	Down
ENSG00000210112	*MT-TM*	1	0	1	0	2.25E-06	-1.19	Down
ENSG00000137818	*RPLP1*	1	0	0	0	2.27E-06	-0.61	Down
ENSG00000111674	*ENO2*	0	0	1	0	2.29E-06	0.74	Up
ENSG00000123505	*AMD1*	0	0	1	0	2.40E-06	-0.57	Down
ENSG00000123240	*OPTN*	1	0	0	0	2.45E-06	-0.71	Down
ENSG00000132938	*MTUS2*	0	0	1	0	2.63E-06	-0.62	Down
ENSG00000161016	*RPL8*	1	0	0	0	2.64E-06	-0.60	Down
ENSG00000137312	*FLOT1*	1	0	0	0	2.84E-06	-0.60	Down
ENSG00000163710	*PCOLCE2*	0	0	1	0	3.25E-06	0.77	Up
ENSG00000198959	*TGM2*	1	1	0	0	3.38E-06	-0.76	Down
ENSG00000186298	*PPP1CC*	1	0	1	0	3.40E-06	-0.84	Down

ENSG00000168610	*STAT3*	1	1	0	0	3.51E-06	-0.91	Down
ENSG00000111452	*ADGRD1*	1	0	1	0	3.54E-06	-0.77	Down
ENSG00000092439	*TRPM7*	0	1	1	0	3.57E-06	-0.65	Down
ENSG00000078804	*TP53INP2*	1	0	0	0	3.64E-06	0.58	Up
ENSG00000196642	*RABL6*	1	0	0	0	3.65E-06	-0.26	Down
ENSG00000188677	*PARVB*	1	0	1	0	3.90E-06	-0.81	Down
ENSG00000143401	*ANP32E*	1	1	0	0	3.94E-06	0.59	Up
ENSG00000167552	*TUBA1A*	1	0	0	0	3.94E-06	0.56	Up
ENSG00000133065	*SLC41A1*	1	0	1	0	4.12E-06	-0.61	Down
ENSG00000089225	*TBX5*	1	0	0	0	4.33E-06	-0.58	Down
ENSG00000175166	*PSMD2*	1	0	0	0	4.44E-06	-0.45	Down
ENSG00000171853	*TRAPPC12*	1	0	0	0	4.51E-06	-0.59	Down
ENSG00000140691	*ARMC5*	1	0	1	0	4.59E-06	0.48	Up
ENSG00000125827	*TMX4*	0	1	0	0	5.10E-06	-0.46	Down
ENSG00000262655	*SPON1*	1	0	0	0	5.10E-06	0.76	Up
ENSG00000104980	*TIMM44*	1	0	1	0	5.12E-06	-0.64	Down
ENSG00000124766	*SOX4*	0	1	0	0	5.44E-06	1.06	Up
ENSG00000117592	*PRDX6*	1	1	0	0	5.47E-06	-0.74	Down
ENSG00000184489	*PTP4A3*	1	0	1	0	5.76E-06	-0.69	Down
ENSG00000173801	*JUP*	1	0	0	0	5.85E-06	-0.63	Down
ENSG00000068976	*PYGM*	1	0	1	0	5.89E-06	-0.74	Down
ENSG00000128791	*TWSG1*	1	0	1	0	6.04E-06	1.12	Up
ENSG00000099998	*GGT5*	1	1	0	0	6.40E-06	-0.95	Down
ENSG00000206560	*ANKRD28*	0	1	0	0	6.51E-06	-0.39	Down
ENSG00000100170	*SLC5A1*	1	0	1	0	6.57E-06	-0.86	Down
ENSG00000163220	*S100A9*	1	0	1	0	6.58E-06	-1.55	Down
ENSG00000131477	*RAMP2*	0	1	0	0	6.59E-06	0.45	Up
ENSG00000125733	*TRIP10*	1	0	0	0	7.05E-06	-0.59	Down
ENSG00000114631	*PODXL2*	0	0	1	0	7.15E-06	1.10	Up
ENSG00000128309	*MPST*	1	1	0	0	7.21E-06	-0.96	Down
ENSG00000267577	*AC010327.3*	1	0	0	0	7.66E-06	1.09	Up
ENSG00000144810	*COL8A1*	0	0	1	0	7.83E-06	0.77	Up
ENSG00000105953	*OGDH*	1	0	1	0	7.85E-06	-0.57	Down
ENSG00000108592	*FTSJ3*	1	0	0	0	7.99E-06	-0.29	Down
ENSG00000053254	*FOXN3*	1	0	1	0	8.09E-06	-0.65	Down
ENSG00000183098	*GPC6*	0	0	1	0	8.09E-06	1.46	Up
ENSG00000171552	*BCL2L1*	0	1	1	0	8.20E-06	-0.74	Down
ENSG00000183072	*NKX2-5*	1	0	0	0	8.22E-06	-0.85	Down
ENSG00000000971	*CFH*	0	0	1	0	8.42E-06	1.14	Up
ENSG00000187840	*EIF4EBP1*	0	0	1	0	8.55E-06	-0.99	Down
ENSG00000113657	*DPYSL3*	0	1	1	0	8.70E-06	0.84	Up
ENSG00000075618	*FSCN1*	0	1	1	0	8.97E-06	0.90	Up
ENSG00000051382	*PIK3CB*	0	1	1	0	8.98E-06	-0.36	Down
ENSG00000135821	*GLUL*	0	1	1	0	8.98E-06	-0.55	Down
ENSG00000107262	*BAG1*	1	0	0	0	1.04E-05	-0.53	Down
ENSG00000235106	*BRD3OS*	1	0	0	0	1.05E-05	0.34	Up
ENSG00000183722	*LHFPL6*	1	1	0	0	1.10E-05	0.54	Up
ENSG00000118523	*CCN2*	0	0	1	0	1.12E-05	1.19	Up
ENSG00000135185	*TMEM243*	1	0	0	0	1.13E-05	0.46	Up
ENSG00000178814	*OPLAH*	0	0	1	0	1.14E-05	-0.48	Down
ENSG00000164338	*UTP15*	1	0	0	0	1.16E-05	-0.51	Down
ENSG00000270885	*RASL10B*	0	0	1	0	1.17E-05	-1.01	Down
ENSG00000117394	*SLC2A1*	1	0	0	0	1.30E-05	-1.06	Down

ENSG00000139289	*PHLDA1*	1	0	0	0	1.31E-05	1.14	Up
ENSG00000160294	*MCM3AP*	1	0	0	0	1.33E-05	-0.44	Down
ENSG00000166313	*APBB1*	1	0	1	0	1.33E-05	-0.57	Down
ENSG00000100605	*ITPK1*	0	1	1	0	1.35E-05	-0.53	Down
ENSG00000262049	*AC139530.2*	1	0	1	0	1.36E-05	0.35	Up
ENSG00000172458	*IL17D*	0	0	1	0	1.42E-05	0.79	Up
ENSG00000153207	*AHCTF1*	0	0	1	0	1.46E-05	-0.58	Down
ENSG00000142973	*CYP4B1*	0	1	1	0	1.49E-05	-1.56	Down
ENSG00000187778	*MCRS1*	1	0	1	0	1.61E-05	-0.77	Down
ENSG00000120820	*GLT8D2*	0	1	0	0	1.63E-05	0.83	Up
ENSG00000156535	*CD109*	0	1	0	0	1.63E-05	-0.56	Down
ENSG00000160801	*PTH1R*	0	0	1	0	1.71E-05	-0.75	Down
ENSG00000178209	*PLEC*	1	0	0	0	1.71E-05	-0.39	Down
ENSG00000073008	*PVR*	1	0	1	0	1.78E-05	-0.93	Down
ENSG00000120699	*EXOSC8*	1	0	0	0	1.88E-05	0.35	Up
ENSG00000175206	*NPPA*	1	0	1	0	1.91E-05	1.86	Up
ENSG00000140350	*ANP32A*	1	0	1	0	1.93E-05	0.42	Up
ENSG00000161011	*SQSTM1*	1	0	1	0	1.95E-05	0.30	Up
ENSG00000211448	*DIO2*	1	1	0	0	1.95E-05	1.30	Up
ENSG00000142541	*RPL13A*	1	0	0	0	1.97E-05	-0.51	Down
ENSG00000236753	*MKLN1-AS*	1	0	0	0	2.00E-05	-0.72	Down
ENSG00000178741	*COX5A*	1	0	1	0	2.07E-05	-0.72	Down
ENSG00000130779	*CLIP1*	1	0	1	0	2.14E-05	-0.78	Down
ENSG00000272288	*AL451165.2*	1	0	1	0	2.30E-05	-0.35	Down
ENSG00000017427	*IGF1*	0	1	0	0	2.44E-05	0.82	Up
ENSG00000173715	*C11orf80*	0	0	1	0	2.61E-05	1.01	Up
ENSG00000116717	*GADD45A*	1	0	0	0	2.67E-05	0.82	Up
ENSG00000134030	*CTIF*	1	0	0	0	2.67E-05	-0.66	Down
ENSG00000132329	*RAMP1*	0	1	0	0	2.74E-05	-0.83	Down
ENSG00000088899	*LZTS3*	0	0	1	0	2.75E-05	-0.51	Down
ENSG00000250479	*CHCHD10*	1	0	0	0	2.81E-05	-0.51	Down
ENSG00000118292	*C1orf54*	1	0	0	0	2.82E-05	0.52	Up
ENSG00000135766	*EGLN1*	0	1	1	0	2.92E-05	-0.46	Down
ENSG00000175287	*PHYHD1*	0	1	1	0	3.03E-05	-0.84	Down
ENSG00000092820	*EZR*	1	1	0	0	3.09E-05	-0.32	Down
ENSG00000122435	*TRMT13*	1	0	0	0	3.09E-05	-0.39	Down
ENSG00000144959	*NCEH1*	1	0	1	0	3.25E-05	-0.69	Down
ENSG00000125378	*BMP4*	0	1	1	0	3.63E-05	0.63	Up
ENSG00000126777	*KTN1*	1	0	0	0	3.76E-05	-0.58	Down
ENSG00000166086	*JAM3*	0	0	1	0	3.89E-05	0.47	Up
ENSG00000133026	*MYH10*	0	0	1	0	3.94E-05	0.66	Up
ENSG00000100823	*APEX1*	1	0	0	0	4.09E-05	-0.64	Down
ENSG00000237172	*B3GNT9*	1	0	1	0	4.09E-05	0.38	Up
ENSG00000106484	*MEST*	1	0	0	0	4.16E-05	0.55	Up
ENSG00000160888	*IER2*	0	1	1	0	4.24E-05	0.83	Up
ENSG00000187642	*PERM1*	1	0	0	0	4.30E-05	-0.65	Down
ENSG00000089157	*RPLP0*	1	0	0	0	4.32E-05	-0.92	Down
ENSG00000104881	*PPP1R13L*	1	0	1	0	4.38E-05	-0.54	Down
ENSG00000011295	*TTC19*	1	0	0	0	4.41E-05	-0.35	Down
ENSG00000214253	*FIS1*	1	0	0	0	4.50E-05	-0.70	Down
ENSG00000166685	*COG1*	1	0	0	0	4.56E-05	-0.19	Down
ENSG00000110492	*MDK*	0	1	1	0	4.82E-05	0.96	Up
ENSG00000131620	*ANO1*	0	1	0	0	4.82E-05	0.79	Up

ENSG00000047457	*CP*	0	0	1	0	4.85E-05	0.77	Up
ENSG00000068878	*PSME4*	0	1	0	0	4.87E-05	-0.54	Down
ENSG00000100628	*ASB2*	1	0	0	0	4.87E-05	-0.79	Down
ENSG00000174444	*RPL4*	1	0	0	0	4.91E-05	-0.53	Down
ENSG00000167671	*UBXN6*	1	0	0	0	4.97E-05	-0.31	Down
ENSG00000114735	*HEMK1*	0	0	1	0	5.02E-05	0.47	Up
ENSG00000055208	*TAB2*	1	0	1	0	5.24E-05	-0.85	Down
ENSG00000134851	*TMEM165*	1	0	0	0	5.27E-05	-0.56	Down
ENSG00000172292	*CERS6*	0	1	0	0	5.27E-05	-0.32	Down
ENSG00000121413	*ZSCAN18*	1	0	1	0	5.28E-05	0.45	Up
ENSG00000056736	*IL17RB*	1	0	1	0	5.29E-05	-1.33	Down
ENSG00000100416	*TRMU*	0	1	1	0	5.89E-05	-0.41	Down
ENSG00000163884	*KLF15*	0	1	1	0	5.89E-05	-0.96	Down
ENSG00000198467	*TPM2*	1	0	0	0	6.33E-05	-0.44	Down
ENSG00000011105	*TSPAN9*	0	0	1	0	6.38E-05	0.55	Up
ENSG00000158246	*TENT5B*	1	0	1	0	6.52E-05	-1.22	Down
ENSG00000134508	*CABLES1*	0	1	0	0	7.06E-05	1.00	Up
ENSG00000072274	*TFRC*	1	1	0	0	7.18E-05	-1.17	Down
ENSG00000113649	*TCERG1*	1	0	1	0	7.38E-05	-0.57	Down
ENSG00000104177	*MYEF2*	0	0	1	0	7.46E-05	0.73	Up
ENSG00000153132	*CLGN*	1	0	0	0	7.56E-05	-0.69	Down
ENSG00000235387	*SPAAR*	0	0	1	0	7.82E-05	-0.71	Down
ENSG00000189058	*APOD*	1	0	0	0	8.18E-05	-1.01	Down
ENSG00000176463	*SLCO3A1*	1	0	0	0	8.36E-05	-0.60	Down
ENSG00000168653	*NDUFS5*	1	0	1	0	8.40E-05	-0.59	Down
ENSG00000118495	*PLAGL1*	0	1	1	0	8.46E-05	0.64	Up
ENSG00000124172	*ATP5F1E*	1	0	0	0	8.49E-05	-0.66	Down
ENSG00000188257	*PLA2G2A*	1	0	0	0	8.91E-05	-1.83	Down
ENSG00000156050	*FAM161B*	1	0	1	0	9.04E-05	0.35	Up
ENSG00000166913	*YWHAB*	1	0	0	0	9.04E-05	0.48	Up
ENSG00000248923	*MTND5P11*	1	0	0	0	9.21E-05	-1.12	Down
ENSG00000231711	*LINC00899*	0	0	1	0	9.25E-05	0.62	Up
ENSG00000134046	*MBD2*	1	1	0	0	9.28E-05	-0.71	Down
ENSG00000132507	*EIF5A*	1	0	0	0	9.36E-05	-0.44	Down
ENSG00000154642	*C21orf91*	1	0	1	0	9.60E-05	1.04	Up
ENSG00000060138	*YBX3*	0	1	0	0	9.70E-05	-0.49	Down
ENSG00000140564	*FURIN*	1	1	0	0	9.81E-05	-0.66	Down
ENSG00000235505	*CASP17P*	1	0	1	0	9.87E-05	1.33	Up
ENSG00000167315	*ACAA2*	1	0	1	0	1.09E-04	-0.80	Down
ENSG00000008513	*ST3GAL1*	1	1	0	0	1.14E-04	-0.58	Down
ENSG00000176971	*FIBIN*	0	0	1	0	1.14E-04	0.86	Up
ENSG00000205726	*ITSN1*	1	0	0	0	1.16E-04	-0.44	Down
ENSG00000160685	*ZBTB7B*	1	0	0	0	1.19E-04	-0.85	Down
ENSG00000071242	*RPS6KA2*	0	1	0	0	1.22E-04	-0.54	Down
ENSG00000163638	*ADAMTS9*	0	1	1	0	1.25E-04	-0.78	Down
ENSG00000152642	*GPD1L*	1	0	1	0	1.26E-04	-0.75	Down
ENSG00000120910	*PPP3CC*	0	1	0	0	1.28E-04	-0.63	Down
ENSG00000205336	*ADGRG1*	0	0	1	0	1.28E-04	-0.27	Down
ENSG00000092054	*MYH7*	1	0	0	0	1.28E-04	-0.45	Down
ENSG00000109339	*MAPK10*	0	1	1	0	1.28E-04	0.76	Up
ENSG00000083845	*RPS5*	1	0	0	0	1.31E-04	-0.71	Down
ENSG00000102466	*FGF14*	1	0	1	0	1.34E-04	0.79	Up
ENSG00000109610	*SOD3*	0	0	1	0	1.36E-04	0.77	Up

ENSG00000118898	*PPL*	0	0	1	0	1.41E-04	-0.48	Down
ENSG00000173641	*HSPB7*	1	0	0	0	1.41E-04	-0.45	Down
ENSG00000173545	*ZNF622*	1	1	0	0	1.45E-04	-0.63	Down
ENSG00000165887	*ANKRD2*	1	0	1	0	1.46E-04	-1.39	Down
ENSG00000136925	*TSTD2*	1	0	0	0	1.47E-04	-0.24	Down
ENSG00000173272	*MZT2A*	1	0	1	0	1.47E-04	-0.69	Down
ENSG00000155096	*AZIN1*	1	0	1	0	1.48E-04	0.55	Up
ENSG00000119650	*IFT43*	0	0	1	0	1.50E-04	0.64	Up
ENSG00000186340	*THBS2*	0	0	1	0	1.57E-04	0.80	Up
ENSG00000128591	*FLNC*	1	0	0	0	1.67E-04	-0.85	Down
ENSG00000142409	*ZNF787*	0	0	1	0	1.74E-04	-0.64	Down
ENSG00000184205	*TSPYL2*	0	1	0	0	1.75E-04	-0.66	Down
ENSG00000187147	*RNF220*	1	0	0	0	1.81E-04	-0.36	Down
ENSG00000137076	*TLN1*	1	0	0	0	1.87E-04	-0.47	Down
ENSG00000161649	*CD300LG*	0	0	1	0	1.87E-04	-0.71	Down
ENSG00000178585	*CTNNBIP1*	0	0	1	0	1.91E-04	-0.82	Down
ENSG00000260807	*AC009041.2*	1	0	1	0	1.98E-04	1.32	Up
ENSG00000137547	*MRPL15*	1	0	0	0	1.99E-04	-0.70	Down
ENSG00000115380	*EFEMP1*	0	1	1	0	2.04E-04	0.90	Up
ENSG00000140829	*DHX38*	0	1	0	0	2.12E-04	-0.41	Down
ENSG00000128918	*ALDH1A2*	0	1	0	0	2.13E-04	0.80	Up
ENSG00000005844	*ITGAL*	0	1	0	0	2.17E-04	0.97	Up
ENSG00000164932	*CTHRC1*	0	1	1	0	2.18E-04	1.44	Up
ENSG00000125743	*SNRPD2*	1	0	0	0	2.19E-04	-0.59	Down
ENSG00000130159	*ECSIT*	1	0	1	0	2.25E-04	-0.59	Down
ENSG00000160959	*LRRC14*	1	0	0	0	2.37E-04	0.36	Up
ENSG00000123096	*SSPN*	0	0	1	0	2.40E-04	0.33	Up
ENSG00000162882	*HAAO*	0	0	1	0	2.49E-04	1.32	Up
ENSG00000124701	*APOBEC2*	1	0	1	0	2.58E-04	-0.69	Down
ENSG00000141965	*FEM1A*	1	0	0	0	2.58E-04	-0.73	Down
ENSG00000173457	*PPP1R14B*	1	0	0	0	2.62E-04	-0.88	Down
ENSG00000186350	*RXRA*	1	0	1	0	2.63E-04	-0.55	Down
ENSG00000138592	*USP8*	1	0	1	0	2.67E-04	-0.37	Down
ENSG00000164050	*PLXNB1*	0	0	1	0	2.67E-04	-0.55	Down
ENSG00000188783	*PRELP*	0	0	1	0	2.72E-04	0.80	Up
ENSG00000170142	*UBE2E1*	1	0	0	0	2.75E-04	-0.29	Down
ENSG00000176597	*B3GNT5*	0	1	1	0	2.76E-04	0.90	Up
ENSG00000144218	*AFF3*	0	1	0	0	2.78E-04	0.71	Up
ENSG00000160113	*NR2F6*	1	0	1	0	2.87E-04	-0.61	Down
ENSG00000141736	*ERBB2*	1	0	0	0	2.91E-04	-0.39	Down
ENSG00000237945	*LINC00649*	1	0	1	0	2.92E-04	-0.64	Down
ENSG00000170962	*PDGFD*	1	0	0	0	3.01E-04	0.97	Up
ENSG00000077942	*FBLN1*	0	1	1	0	3.01E-04	1.03	Up
ENSG00000176485	*PLA2G16*	1	0	0	0	3.25E-04	-0.73	Down
ENSG00000224699	*LAMTOR5-AS1*	1	0	0	0	3.30E-04	-0.77	Down
ENSG00000146147	*MLIP*	1	0	1	0	3.48E-04	-0.77	Down
ENSG00000143515	*ATP8B2*	1	0	1	0	3.63E-04	0.87	Up
ENSG00000143995	*MEIS1*	0	1	0	0	3.64E-04	-0.50	Down
ENSG00000066855	*MTFR1*	1	0	1	0	3.66E-04	-0.49	Down
ENSG00000187079	*TEAD1*	1	0	1	0	3.81E-04	-0.73	Down
ENSG00000107036	*RIC1*	1	0	0	0	3.81E-04	-0.46	Down
ENSG00000171608	*PIK3CD*	1	0	0	0	3.84E-04	0.36	Up
ENSG00000130300	*PLVAP*	0	0	1	0	3.94E-04	0.69	Up

ENSG00000014216	*CAPN1*	1	0	0	0	4.08E-04	-0.41	Down
ENSG00000159167	*STC1*	0	0	1	0	4.09E-04	1.41	Up
ENSG00000168309	*FAM107A*	0	0	1	0	4.09E-04	-0.62	Down
ENSG00000158417	*EIF5B*	1	0	0	0	4.10E-04	-0.51	Down
ENSG00000161381	*PLXDC1*	0	1	1	0	4.12E-04	0.65	Up
ENSG00000172164	*SNTB1*	0	1	0	0	4.25E-04	0.55	Up
ENSG00000119242	*CCDC92*	1	0	0	0	4.63E-04	-0.48	Down
ENSG00000215375	*MYL5*	1	0	0	0	4.63E-04	-0.27	Down
ENSG00000170734	*POLH*	1	0	0	0	4.71E-04	0.45	Up
ENSG00000079215	*SLC1A3*	0	1	1	0	4.75E-04	-0.47	Down
ENSG00000100106	*TRIOBP*	1	0	0	0	4.80E-04	-0.48	Down
ENSG00000182162	*P2RY8*	1	1	0	0	4.80E-04	1.21	Up
ENSG00000176658	*MYO1D*	0	0	1	0	4.82E-04	0.82	Up
ENSG00000166484	*MAPK7*	1	0	0	0	4.83E-04	0.62	Up
ENSG00000127418	*FGFRL1*	1	1	0	0	4.83E-04	-0.77	Down
ENSG00000131899	*LLGL1*	1	0	0	0	4.99E-04	0.44	Up
ENSG00000225526	*MKRN2OS*	0	0	1	0	5.00E-04	1.28	Up
ENSG00000110811	*P3H3*	0	1	0	0	5.05E-04	0.59	Up
ENSG00000095203	*EPB41L4B*	0	0	1	0	5.11E-04	-0.73	Down
ENSG00000160949	*TONSL*	1	0	0	0	5.15E-04	0.52	Up
ENSG00000113083	*LOX*	1	0	1	0	5.16E-04	0.85	Up
ENSG00000137309	*HMGA1*	1	0	0	0	5.28E-04	-0.57	Down
ENSG00000101210	*EEF1A2*	1	0	0	0	5.33E-04	-0.35	Down
ENSG00000151503	*NCAPD3*	1	0	0	0	5.43E-04	0.22	Up
ENSG00000167106	*FAM102A*	0	0	1	0	5.47E-04	0.69	Up
ENSG00000111341	*MGP*	0	0	1	0	5.63E-04	0.62	Up
ENSG00000147679	*UTP23*	1	0	0	0	5.63E-04	-0.25	Down
ENSG00000072864	*NDE1*	1	0	0	0	5.63E-04	0.56	Up
ENSG00000177098	*SCN4B*	0	1	0	0	5.72E-04	0.62	Up
ENSG00000137776	*SLTM*	1	0	0	0	5.74E-04	-0.49	Down
ENSG00000162191	*UBXN1*	1	0	0	0	5.93E-04	-0.56	Down
ENSG00000197893	*NRAP*	1	0	0	0	5.98E-04	-0.65	Down
ENSG00000198771	*RCSD1*	1	0	1	0	5.98E-04	-0.46	Down
ENSG00000124588	*NQO2*	1	0	1	0	6.00E-04	-0.73	Down
ENSG00000103335	*PIEZO1*	1	0	0	0	6.09E-04	0.36	Up
ENSG00000188176	*SMTNL2*	0	0	1	0	6.17E-04	-0.84	Down
ENSG00000100023	*PPIL2*	1	0	0	0	6.24E-04	0.31	Up
ENSG00000105357	*MYH14*	1	0	0	0	6.40E-04	-0.54	Down
ENSG00000104853	*CLPTM1*	1	0	0	0	6.65E-04	-0.37	Down
ENSG00000112306	*RPS12*	1	0	0	0	6.68E-04	-0.86	Down
ENSG00000167815	*PRDX2*	1	0	0	0	6.78E-04	-0.66	Down
ENSG00000144034	*TPRKB*	0	0	1	0	6.89E-04	-0.38	Down
ENSG00000142188	*TMEM50B*	1	0	0	0	7.07E-04	0.29	Up
ENSG00000165795	*NDRG2*	0	0	1	0	7.15E-04	-0.29	Down
ENSG00000120594	*PLXDC2*	0	0	1	0	7.18E-04	0.60	Up
ENSG00000140199	*SLC12A6*	1	0	0	0	7.26E-04	-0.25	Down
ENSG00000277494	*GPIHBP1*	0	0	1	0	7.52E-04	-0.60	Down
ENSG00000235535	*TRDN-AS1*	1	0	0	0	7.85E-04	-0.45	Down
ENSG00000197912	*SPG7*	1	0	1	0	8.00E-04	-0.41	Down
ENSG00000171517	*LPAR3*	0	0	0	0	8.64E-04	-1.16	Down
ENSG00000163513	*TGFBR2*	0	0	1	0	8.77E-04	-0.41	Down
ENSG00000136111	*TBC1D4*	1	0	1	0	8.77E-04	-0.64	Down
ENSG00000142279	*WTIP*	0	1	1	0	8.85E-04	0.80	Up

ENSG00000166002	*SMCO4*	1	0	0	0	8.86E-04	-0.82	Down
ENSG00000113555	*PCDH12*	0	1	0	0	8.97E-04	0.48	Up
ENSG00000197448	*GSTK1*	1	0	1	0	9.05E-04	-0.40	Down
ENSG00000101421	*CHMP4B*	1	0	0	0	9.14E-04	-0.51	Down
ENSG00000108387	*SEPT4*	1	1	0	0	9.30E-04	-0.65	Down
ENSG00000106538	*RARRES2*	0	1	0	0	9.31E-04	1.06	Up
ENSG00000170921	*TANC2*	0	0	1	0	9.48E-04	0.43	Up
ENSG00000126749	*EMG1*	0	0	1	0	9.59E-04	-0.30	Down
ENSG00000245293	*AC096564.1*	1	0	0	0	9.76E-04	-0.68	Down
ENSG00000142856	*ITGB3BP*	0	0	1	0	1.01E-03	-0.40	Down
ENSG00000116473	*RAP1A*	0	1	0	0	1.02E-03	-0.33	Down
ENSG00000172057	*ORMDL3*	0	0	1	0	1.02E-03	0.41	Up
ENSG00000005436	*GCFC2*	0	1	1	0	1.03E-03	-0.38	Down
ENSG00000160539	*PLPP7*	1	0	1	0	1.05E-03	-0.66	Down
ENSG00000159199	*ATP5MC1*	1	0	1	0	1.05E-03	-0.90	Down
ENSG00000084754	*HADHA*	0	0	1	0	1.06E-03	-0.39	Down
ENSG00000169184	*MN1*	0	0	1	0	1.07E-03	0.70	Up
ENSG00000100211	*CBY1*	1	0	0	0	1.07E-03	0.29	Up
ENSG00000163328	*GPR155*	0	0	1	0	1.08E-03	-0.40	Down
ENSG00000102225	*CDK16*	1	0	0	0	1.09E-03	-0.54	Down
ENSG00000119285	*HEATR1*	1	0	0	0	1.09E-03	-0.31	Down
ENSG00000087245	*MMP2*	0	1	0	0	1.10E-03	0.71	Up
ENSG00000119487	*MAPKAP1*	0	0	1	0	1.10E-03	-0.42	Down
ENSG00000186174	*BCL9L*	1	0	0	0	1.11E-03	-0.74	Down
ENSG00000100296	*THOC5*	1	1	0	0	1.14E-03	-0.68	Down
ENSG00000175110	*MRPS22*	1	0	0	0	1.14E-03	-0.21	Down
ENSG00000140941	*MAP1LC3B*	1	0	0	0	1.18E-03	-0.53	Down
ENSG00000126581	*BECN1*	1	0	0	0	1.18E-03	-0.57	Down
ENSG00000130725	*UBE2M*	1	0	0	0	1.18E-03	-0.51	Down
ENSG00000072840	*EVC*	0	1	0	0	1.25E-03	0.66	Up
ENSG00000077684	*JADE1*	0	1	0	0	1.28E-03	-0.27	Down
ENSG00000120053	*GOT1*	1	0	0	0	1.28E-03	-0.54	Down
ENSG00000197077	*KIAA1671*	1	1	0	0	1.28E-03	0.65	Up
ENSG00000178605	*GTPBP6*	0	0	1	0	1.28E-03	-0.47	Down
ENSG00000124562	*SNRPC*	1	0	1	0	1.29E-03	-0.63	Down
ENSG00000176407	*KCMF1*	0	1	0	0	1.29E-03	-0.42	Down
ENSG00000228794	*LINC01128*	0	0	1	0	1.30E-03	-0.37	Down
ENSG00000110092	*CCND1*	0	0	1	0	1.30E-03	0.35	Up
ENSG00000164142	*FAM160A1*	0	1	0	0	1.32E-03	-0.49	Down
ENSG00000141447	*OSBPL1A*	1	0	1	0	1.32E-03	-0.40	Down
ENSG00000172349	*IL16*	0	1	0	0	1.32E-03	0.61	Up
ENSG00000123144	*TRIR*	1	0	0	0	1.33E-03	-0.53	Down
ENSG00000136870	*ZNF189*	1	0	0	0	1.33E-03	-1.05	Down
ENSG00000072163	*LIMS2*	0	0	1	0	1.36E-03	-0.25	Down
ENSG00000142937	*RPS8*	1	0	0	0	1.47E-03	-0.50	Down
ENSG00000111728	*ST8SIA1*	0	1	0	0	1.53E-03	0.89	Up
ENSG00000175220	*ARHGAP1*	0	0	1	0	1.59E-03	0.43	Up
ENSG00000221983	*UBA52*	1	0	0	0	1.61E-03	-0.69	Down
ENSG00000174502	*SLC26A9*	0	0	1	0	1.63E-03	-0.55	Down
ENSG00000134755	*DSC2*	0	0	1	0	1.66E-03	-0.58	Down
ENSG00000005448	*WDR54*	1	0	0	0	1.70E-03	0.72	Up
ENSG00000099875	*MKNK2*	1	0	0	0	1.74E-03	-0.75	Down
ENSG00000146242	*TPBG*	0	1	0	0	1.75E-03	0.73	Up

ENSG00000127663	*KDM4B*	1	0	1	0	1.76E-03	-0.55	Down
ENSG00000186591	*UBE2H*	1	0	1	0	1.76E-03	0.42	Up
ENSG00000148498	*PARD3*	0	1	0	0	1.78E-03	-0.50	Down
ENSG00000256043	*CTSO*	0	1	0	0	1.81E-03	0.61	Up
ENSG00000070778	*AL162171.1*	0	1	0	0	1.82E-03	0.35	Up
ENSG00000089199	*CHGB*	1	0	0	0	1.83E-03	-1.55	Down
ENSG00000116260	*QSOX1*	1	1	0	0	1.85E-03	-0.77	Down
ENSG00000196531	*NACA*	1	0	0	0	1.86E-03	-0.53	Down
ENSG00000178982	*EIF3K*	1	0	0	0	1.87E-03	-0.60	Down
ENSG00000100393	*EP300*	1	0	0	0	1.90E-03	-0.43	Down
ENSG00000007080	*CCDC124*	1	0	0	0	1.90E-03	-0.43	Down
ENSG00000203734	*ECT2L*	1	0	1	0	1.91E-03	1.07	Up
ENSG00000074219	*TEAD2*	0	0	1	0	1.92E-03	-0.51	Down
ENSG00000188735	*TMEM120B*	1	0	0	0	1.92E-03	-0.49	Down
ENSG00000198932	*GPRASP1*	0	0	1	0	1.93E-03	0.66	Up
ENSG00000121281	*ADCY7*	1	0	0	0	2.00E-03	0.56	Up
ENSG00000104635	*SLC39A14*	0	1	0	0	2.04E-03	-0.57	Down
ENSG00000079805	*DNM2*	0	0	1	0	2.08E-03	-0.24	Down
ENSG00000182154	*MRPL41*	1	0	1	0	2.11E-03	-0.58	Down
ENSG00000149970	*CNKSR2*	1	0	1	0	2.12E-03	0.92	Up
ENSG00000184076	*UQCR10*	1	0	0	0	2.19E-03	-0.55	Down
ENSG00000178607	*ERN1*	0	0	0	0	2.24E-03	0.88	Up
ENSG00000245105	*A2M-AS1*	1	0	0	0	2.25E-03	0.33	Up
ENSG00000143149	*ALDH9A1*	1	0	0	0	2.29E-03	0.53	Up
ENSG00000197622	*CDC42SE1*	1	0	1	0	2.36E-03	0.38	Up
ENSG00000031081	*ARHGAP31*	0	0	1	0	2.40E-03	-0.43	Down
ENSG00000110697	*PITPNM1*	1	0	0	0	2.42E-03	-0.72	Down
ENSG00000204463	*BAG6*	1	0	0	0	2.48E-03	-0.40	Down
ENSG00000172831	*CES2*	1	0	0	0	2.53E-03	-0.62	Down
ENSG00000128731	*HERC2*	1	0	0	0	2.65E-03	-0.54	Down
ENSG00000174231	*PRPF8*	1	0	0	0	2.76E-03	-0.41	Down
ENSG00000018280	*SLC11A1*	0	1	0	0	2.84E-03	-1.31	Down
ENSG00000130560	*UBAC1*	1	0	0	0	2.85E-03	-0.60	Down
ENSG00000171863	*RPS7*	1	0	0	0	2.94E-03	-0.93	Down
ENSG00000176946	*THAP4*	1	0	1	0	2.94E-03	-0.51	Down
ENSG00000145725	*PPIP5K2*	0	0	1	0	2.98E-03	-0.78	Down
ENSG00000197852	*INKA2*	0	1	1	0	2.98E-03	-0.43	Down
ENSG00000121022	*COPS5*	1	0	0	0	3.04E-03	-0.49	Down
ENSG00000137752	*CASP1*	0	1	1	0	3.05E-03	0.80	Up
ENSG00000235750	*KIAA0040*	0	0	1	0	3.06E-03	-0.62	Down
ENSG00000103363	*ELOB*	1	0	0	0	3.08E-03	-0.59	Down
ENSG00000184207	*PGP*	0	0	1	0	3.12E-03	-0.50	Down
ENSG00000241644	*INMT*	0	1	0	0	3.16E-03	0.72	Up
ENSG00000048707	*VPS13D*	0	0	0	0	3.17E-03	-0.60	Down
ENSG00000127616	*SMARCA4*	1	0	0	0	3.17E-03	-0.38	Down
ENSG00000175550	*DRAP1*	1	0	0	0	3.18E-03	-0.38	Down
ENSG00000179604	*CDC42EP4*	0	0	0	0	3.18E-03	-0.66	Down
ENSG00000114166	*KAT2B*	1	0	1	0	3.30E-03	-0.58	Down
ENSG00000138709	*LARP1B*	0	0	1	0	3.41E-03	-0.52	Down
ENSG00000072832	*CRMP1*	0	0	0	0	3.42E-03	0.75	Up
ENSG00000223768	*LINC00205*	0	0	1	0	3.49E-03	0.65	Up
ENSG00000151092	*NGLY1*	0	0	1	0	3.52E-03	-0.43	Down
ENSG00000000457	*SCYL3*	1	0	0	0	3.55E-03	-0.30	Down

ENSG00000099968	*BCL2L13*	0	1	0	0	3.62E-03	-0.41	Down
ENSG00000204851	*PNMA8B*	1	0	0	0	3.65E-03	0.89	Up
ENSG00000135597	*REPS1*	1	0	0	0	3.69E-03	0.49	Up
ENSG00000081803	*CADPS2*	0	0	1	0	3.74E-03	-0.73	Down
ENSG00000164949	*GEM*	0	0	1	0	3.78E-03	0.90	Up
ENSG00000138758	*SEPT11*	0	1	0	0	3.83E-03	0.45	Up
ENSG00000096968	*JAK2*	1	0	0	0	3.87E-03	0.82	Up
ENSG00000169855	*ROBO1*	0	1	0	0	3.94E-03	0.59	Up
ENSG00000188290	*HES4*	1	1	0	0	4.00E-03	0.57	Up
ENSG00000159251	*ACTC1*	0	0	1	0	4.01E-03	-0.45	Down
ENSG00000160075	*SSU72*	0	0	1	0	4.06E-03	-0.45	Down
ENSG00000173281	*PPP1R3B*	1	0	1	0	4.07E-03	-0.47	Down
ENSG00000079739	*PGM1*	0	0	1	0	4.09E-03	-0.33	Down
ENSG00000260186	*LINC02137*	0	0	1	0	4.18E-03	-1.08	Down
ENSG00000226900	*AL451069.1*	1	0	1	0	4.23E-03	-1.12	Down
ENSG00000115241	*PPM1G*	1	0	0	0	4.27E-03	-0.45	Down
ENSG00000155659	*VSIG4*	0	1	0	0	4.27E-03	-1.15	Down
ENSG00000104765	*BNIP3L*	1	0	0	0	4.60E-03	0.49	Up
ENSG00000103404	*USP31*	0	1	0	0	4.62E-03	-0.53	Down
ENSG00000148356	*LRSAM1*	0	0	1	0	4.77E-03	-0.58	Down
ENSG00000100105	*PATZ1*	0	0	1	0	4.78E-03	0.38	Up
ENSG00000196504	*PRPF40A*	1	0	0	0	4.85E-03	-0.34	Down
ENSG00000147155	*EBP*	0	1	0	0	4.90E-03	-0.70	Down
ENSG00000218283	*MORF4L1P1*	1	0	0	0	4.94E-03	-0.74	Down
ENSG00000105516	*DBP*	0	1	0	0	4.95E-03	0.67	Up
ENSG00000115317	*HTRA2*	1	0	0	0	4.97E-03	0.24	Up
ENSG00000165916	*PSMC3*	1	0	0	0	5.01E-03	-0.50	Down
ENSG00000184209	*SNRNP35*	1	0	0	0	5.01E-03	-0.29	Down
ENSG00000061676	*NCKAP1*	0	1	0	0	5.05E-03	-0.24	Down
ENSG00000007402	*CACNA2D2*	1	0	0	0	5.10E-03	0.59	Up
ENSG00000198796	*ALPK2*	1	0	0	0	5.10E-03	-0.44	Down
ENSG00000178573	*MAF*	0	1	0	0	5.24E-03	0.54	Up
ENSG00000164733	*CTSB*	1	1	0	0	5.29E-03	-0.36	Down
ENSG00000144824	*PHLDB2*	0	1	0	0	5.32E-03	0.36	Up
ENSG00000022840	*RNF10*	0	1	0	0	5.36E-03	-0.37	Down
ENSG00000006007	*GDE1*	1	0	0	0	5.40E-03	-0.59	Down
ENSG00000170522	*ELOVL6*	1	0	1	0	5.40E-03	0.81	Up
ENSG00000085760	*MTIF2*	1	0	0	0	5.42E-03	-0.62	Down
ENSG00000141449	*GREB1L*	0	1	0	0	5.54E-03	-0.57	Down
ENSG00000126858	*RHOT1*	0	0	1	0	5.62E-03	-0.39	Down
ENSG00000161981	*SNRNP25*	1	0	1	0	5.76E-03	-0.35	Down
ENSG00000120907	*ADRA1A*	1	0	0	0	5.83E-03	-0.67	Down
ENSG00000182541	*LIMK2*	0	0	1	0	5.89E-03	0.40	Up
ENSG00000138495	*COX17*	1	0	1	0	5.91E-03	-0.43	Down
ENSG00000116132	*PRRX1*	1	0	1	0	5.94E-03	0.56	Up
ENSG00000183873	*SCN5A*	1	0	0	0	5.97E-03	-0.54	Down
ENSG00000134531	*EMP1*	1	0	0	0	6.10E-03	0.79	Up
ENSG00000243156	*MICAL3*	1	0	0	0	6.12E-03	-0.50	Down
ENSG00000137941	*TTLL7*	0	0	1	0	6.14E-03	0.76	Up
ENSG00000130382	*MLLT1*	1	0	0	0	6.26E-03	-0.63	Down
ENSG00000163788	*SNRK*	1	0	0	0	6.26E-03	0.37	Up
ENSG00000196954	*CASP4*	1	0	0	0	6.41E-03	0.84	Up
ENSG00000140986	*RPL3L*	1	0	0	0	6.47E-03	-0.44	Down

ENSG00000162458	*FBLIM1*	0	0	0	0	6.49E-03	-0.79	Down
ENSG00000156650	*KAT6B*	1	0	0	0	6.72E-03	-0.38	Down
ENSG00000175463	*TBC1D10C*	1	1	0	0	6.73E-03	1.03	Up
ENSG00000171310	*CHST11*	0	0	1	0	6.80E-03	0.69	Up
ENSG00000117114	*ADGRL2*	1	0	1	0	6.80E-03	-0.74	Down
ENSG00000122033	*MTIF3*	0	0	1	0	6.85E-03	-0.33	Down
ENSG00000175581	*MRPL48*	0	0	1	0	6.86E-03	-0.44	Down
ENSG00000178307	*TMEM11*	0	0	0	0	6.86E-03	-0.49	Down
ENSG00000115641	*FHL2*	1	0	0	0	6.88E-03	-0.63	Down
ENSG00000050405	*LIMA1*	0	1	0	0	6.92E-03	0.63	Up
ENSG00000213190	*MLLT11*	0	0	1	0	6.94E-03	0.49	Up
ENSG00000110422	*HIPK3*	0	0	1	0	7.20E-03	-0.38	Down
ENSG00000129351	*ILF3*	1	0	0	0	7.30E-03	-0.41	Down
ENSG00000166441	*RPL27A*	1	0	0	0	7.31E-03	-0.30	Down
ENSG00000132589	*FLOT2*	1	0	0	0	7.47E-03	-0.72	Down
ENSG00000131943	*C19orf12*	0	0	1	0	7.48E-03	-0.33	Down
ENSG00000166278	*C2*	0	0	0	0	7.49E-03	1.02	Up
ENSG00000149212	*SESN3*	0	0	1	0	7.60E-03	0.71	Up
ENSG00000107738	*VSIR*	0	0	1	0	7.74E-03	-0.41	Down
ENSG00000131462	*TUBG1*	1	0	1	0	7.76E-03	-0.77	Down
ENSG00000165458	*INPPL1*	1	0	0	0	7.80E-03	-0.52	Down
ENSG00000186184	*POLR1D*	1	0	0	0	7.83E-03	-0.57	Down
ENSG00000148303	*RPL7A*	1	0	0	0	7.88E-03	-0.48	Down
ENSG00000100401	*RANGAP1*	1	0	0	0	7.88E-03	-0.48	Down
ENSG00000163382	*NAXE*	0	0	1	0	7.95E-03	-0.40	Down
ENSG00000130640	*TUBGCP2*	1	0	0	0	8.14E-03	-0.20	Down
ENSG00000121057	*AKAP1*	0	0	1	0	8.15E-03	-0.52	Down
ENSG00000125753	*VASP*	1	0	0	0	8.16E-03	-0.59	Down
ENSG00000228526	*MIR34AHG*	0	0	0	0	8.17E-03	0.97	Up
ENSG00000140009	*ESR2*	1	0	0	0	8.22E-03	-0.78	Down
ENSG00000152284	*TCF7L1*	0	1	0	0	8.24E-03	0.45	Up
ENSG00000178878	*APOLD1*	1	0	0	0	8.24E-03	0.95	Up
ENSG00000165775	*FUNDC2*	0	0	1	0	8.26E-03	-0.35	Down
ENSG00000184007	*PTP4A2*	0	0	0	0	8.29E-03	-0.40	Down
ENSG00000176222	*ZNF404*	1	0	0	0	8.32E-03	0.53	Up
ENSG00000151883	*PARP8*	1	0	0	0	8.38E-03	0.49	Up
ENSG00000132361	*CLUH*	1	0	0	0	8.42E-03	-0.26	Down
ENSG00000160818	*GPATCH4*	0	0	0	0	8.45E-03	-0.48	Down
ENSG00000204397	*CARD16*	1	0	0	0	8.48E-03	1.40	Up
ENSG00000244486	*SCARF2*	0	0	1	0	8.49E-03	0.84	Up
ENSG00000158321	*AUTS2*	0	1	0	0	8.50E-03	0.36	Up
ENSG00000100949	*RABGGTA*	0	0	0	0	8.63E-03	-0.38	Down
ENSG00000132718	*SYT11*	1	0	0	0	8.67E-03	0.68	Up
ENSG00000198663	*C6orf89*	1	0	0	0	8.79E-03	-0.38	Down
ENSG00000120549	*KIAA1217*	0	0	1	0	8.81E-03	-0.45	Down
ENSG00000176472	*ZNF575*	1	0	0	0	8.99E-03	0.36	Up
ENSG00000059145	*UNKL*	1	0	0	0	9.00E-03	0.32	Up
ENSG00000173113	*TRMT112*	1	0	0	0	9.04E-03	-0.22	Down
ENSG00000205339	*IPO7*	0	0	1	0	9.09E-03	-0.50	Down
ENSG00000100564	*PIGH*	1	0	0	0	9.14E-03	-0.44	Down
ENSG00000125656	*CLPP*	0	0	1	0	9.19E-03	-0.61	Down
ENSG00000105186	*ANKRD27*	1	0	0	0	9.24E-03	-0.38	Down
ENSG00000255248	*MIR100HG*	0	0	0	0	9.29E-03	0.35	Up

ENSG00000163110	*PDLIM5*	1	0	0	0	9.31E-03	-0.40	Down
ENSG00000153563	*CD8A*	0	1	0	0	9.38E-03	1.43	Up
ENSG00000003402	*CFLAR*	0	0	1	0	9.48E-03	-0.32	Down
ENSG00000165028	*NIPSNAP3B*	0	0	0	0	9.48E-03	-0.52	Down
ENSG00000226950	*DANCR*	0	0	1	0	9.72E-03	-0.47	Down
ENSG00000166289	*PLEKHF1*	0	0	1	0	9.75E-03	-0.73	Down
ENSG00000138622	*HCN4*	1	0	0	0	9.89E-03	-1.20	Down
ENSG00000094975	*SUCO*	0	0	1	0	9.95E-03	-0.52	Down
ENSG00000257261	*AC008014.1*	0	0	1	0	9.96E-03	-0.71	Down
ENSG00000133678	*TMEM254*	0	0	0	0	9.98E-03	0.63	Up
ENSG00000172201	*ID4*	0	0	1	0	1.00E-02	1.11	Up
ENSG00000172348	*RCAN2*	0	1	0	0	1.01E-02	-0.31	Down
ENSG00000128272	*ATF4*	0	0	0	0	1.02E-02	-0.55	Down
ENSG00000164442	*CITED2*	1	0	0	0	1.04E-02	-0.56	Down
ENSG00000115084	*SLC35F5*	1	0	0	0	1.04E-02	-0.62	Down
ENSG00000095321	*CRAT*	1	0	0	0	1.04E-02	-0.63	Down
ENSG00000184983	*NDUFA6*	1	0	0	0	1.05E-02	-0.40	Down
ENSG00000060339	*CCAR1*	1	0	0	0	1.06E-02	-0.64	Down
ENSG00000147677	*EIF3H*	1	0	0	0	1.06E-02	-0.24	Down
ENSG00000261115	*TMEM178B*	0	0	1	0	1.06E-02	-0.55	Down
ENSG00000006576	*PHTF2*	0	1	0	0	1.07E-02	-0.61	Down
ENSG00000164023	*SGMS2*	1	0	0	0	1.09E-02	-0.30	Down
ENSG00000198874	*TYW1*	1	0	0	0	1.10E-02	0.21	Up
ENSG00000180644	*PRF1*	0	1	0	0	1.12E-02	1.21	Up
ENSG00000173638	*SLC19A1*	1	0	0	0	1.12E-02	0.39	Up
ENSG00000118971	*CCND2*	1	0	0	0	1.12E-02	0.48	Up
ENSG00000103342	*GSPT1*	0	0	0	0	1.13E-02	-0.33	Down
ENSG00000123124	*WWP1*	0	0	1	0	1.14E-02	-0.34	Down
ENSG00000240694	*PNMA2*	0	1	0	0	1.15E-02	0.78	Up
ENSG00000176720	*BOK*	0	0	1	0	1.16E-02	0.72	Up
ENSG00000198793	*MTOR*	1	1	0	0	1.17E-02	-0.45	Down
ENSG00000070010	*UFD1*	1	0	0	0	1.18E-02	-0.36	Down
ENSG00000069702	*TGFBR3*	0	0	1	0	1.19E-02	-0.69	Down
ENSG00000122359	*ANXA11*	1	0	0	0	1.21E-02	-0.41	Down
ENSG00000172725	*CORO1B*	1	0	0	0	1.22E-02	0.53	Up
ENSG00000130312	*MRPL34*	0	0	1	0	1.26E-02	-0.28	Down
ENSG00000163866	*SMIM12*	0	0	0	0	1.27E-02	-0.46	Down
ENSG00000253304	*TMEM200B*	0	0	1	0	1.27E-02	0.50	Up
ENSG00000099785	*MARCH2*	1	0	0	0	1.27E-02	-0.78	Down
ENSG00000124702	*KLHDC3*	0	0	1	0	1.28E-02	-0.31	Down
ENSG00000198355	*PIM3*	0	0	0	0	1.29E-02	-0.61	Down
ENSG00000170270	*GON7*	0	0	1	0	1.30E-02	-0.49	Down
ENSG00000177374	*HIC1*	1	1	0	0	1.31E-02	0.38	Up
ENSG00000163399	*ATP1A1*	0	0	0	0	1.32E-02	-0.47	Down
ENSG00000160862	*AZGP1*	0	0	0	0	1.33E-02	-0.85	Down
ENSG00000135617	*PRADC1*	1	0	0	0	1.37E-02	-0.63	Down
ENSG00000114686	*MRPL3*	0	0	1	0	1.38E-02	-0.61	Down
ENSG00000213722	*DDAH2*	0	0	1	0	1.38E-02	0.39	Up
ENSG00000108797	*CNTNAP1*	1	0	0	0	1.41E-02	0.52	Up
ENSG00000146592	*CREB5*	0	1	0	0	1.42E-02	0.76	Up
ENSG00000117616	*RSRP1*	0	0	1	0	1.42E-02	0.43	Up
ENSG00000180758	*GPR157*	0	0	0	0	1.42E-02	-0.73	Down
ENSG00000188549	*CCDC9B*	1	0	0	0	1.42E-02	-0.38	Down

ENSG00000185104	*FAF1*	1	0	0	0	1.43E-02	-0.70	Down
ENSG00000150457	*LATS2*	1	0	0	0	1.43E-02	0.57	Up
ENSG00000087274	*ADD1*	1	0	0	0	1.44E-02	-0.39	Down
ENSG00000107263	*RAPGEF1*	1	0	0	0	1.46E-02	-0.45	Down
ENSG00000132613	*MTSS1L*	1	0	0	0	1.46E-02	-0.43	Down
ENSG00000151729	*SLC25A4*	1	0	1	0	1.48E-02	-0.37	Down
ENSG00000131697	*NPHP4*	1	0	0	0	1.48E-02	0.89	Up
ENSG00000135074	*ADAM19*	1	0	0	0	1.51E-02	0.65	Up
ENSG00000161791	*FMNL3*	0	0	1	0	1.51E-02	0.36	Up
ENSG00000115840	*SLC25A12*	0	0	1	0	1.53E-02	-0.44	Down
ENSG00000115461	*IGFBP5*	0	0	0	0	1.53E-02	0.48	Up
ENSG00000113916	*BCL6*	0	0	1	0	1.55E-02	-0.54	Down
ENSG00000165269	*AQP7*	1	0	1	0	1.59E-02	-0.38	Down
ENSG00000181896	*ZNF101*	1	0	0	0	1.59E-02	0.64	Up
ENSG00000176903	*PNMA1*	0	0	1	0	1.60E-02	0.50	Up
ENSG00000170871	*KIAA0232*	1	0	0	0	1.67E-02	-0.52	Down
ENSG00000160216	*AGPAT3*	1	0	0	0	1.70E-02	-0.53	Down
ENSG00000164736	*SOX17*	1	0	0	0	1.72E-02	0.86	Up
ENSG00000277791	*PSMB3*	1	0	0	0	1.73E-02	-0.66	Down
ENSG00000152700	*SAR1B*	1	0	0	0	1.73E-02	-0.38	Down
ENSG00000167549	*CORO6*	0	0	1	0	1.75E-02	-0.43	Down
ENSG00000237807	*AC022034.1*	0	0	1	0	1.75E-02	-0.81	Down
ENSG00000147100	*SLC16A2*	0	1	0	0	1.76E-02	0.55	Up
ENSG00000037280	*FLT4*	0	0	0	0	1.76E-02	0.48	Up
ENSG00000148229	*POLE3*	1	0	0	0	1.77E-02	-0.49	Down
ENSG00000248449	*PCDHGB8P*	0	0	1	0	1.78E-02	-0.77	Down
ENSG00000135272	*MDFIC*	0	0	0	0	1.78E-02	0.69	Up
ENSG00000122965	*RBM19*	1	0	0	0	1.78E-02	-0.56	Down
ENSG00000155115	*GTF3C6*	1	0	0	0	1.78E-02	-0.48	Down
ENSG00000182179	*UBA7*	0	0	1	0	1.80E-02	0.53	Up
ENSG00000165886	*UBTD1*	0	0	1	0	1.81E-02	-0.59	Down
ENSG00000259972	*AC009120.2*	1	0	0	0	1.82E-02	-0.35	Down
ENSG00000131781	*FMO5*	0	0	0	0	1.83E-02	-0.73	Down
ENSG00000073792	*IGF2BP2*	0	1	0	0	1.84E-02	-0.73	Down
ENSG00000104904	*OAZ1*	1	0	0	0	1.84E-02	-0.50	Down
ENSG00000139567	*ACVRL1*	0	0	1	0	1.84E-02	-0.46	Down
ENSG00000100711	*ZFYVE21*	0	0	1	0	1.85E-02	0.34	Up
ENSG00000131844	*MCCC2*	1	0	1	0	1.86E-02	-0.47	Down
ENSG00000101400	*SNTA1*	1	0	0	0	1.87E-02	-0.45	Down
ENSG00000134684	*YARS*	0	1	0	0	1.87E-02	-0.33	Down
ENSG00000243147	*MRPL33*	0	0	1	0	1.88E-02	-0.55	Down
ENSG00000168461	*RAB31*	0	0	1	0	1.88E-02	0.58	Up
ENSG00000099889	*ARVCF*	0	1	0	0	1.92E-02	0.50	Up
ENSG00000184887	*BTBD6*	0	0	0	0	1.93E-02	-0.53	Down
ENSG00000107338	*SHB*	0	0	1	0	1.93E-02	0.58	Up
ENSG00000135749	*PCNX2*	0	0	1	0	1.96E-02	0.77	Up
ENSG00000197653	*DNAH10*	1	0	0	0	1.96E-02	-0.91	Down
ENSG00000048162	*NOP16*	1	0	0	0	1.98E-02	-0.34	Down
ENSG00000177600	*RPLP2*	1	0	0	0	1.99E-02	-0.61	Down
ENSG00000178980	*SELENOW*	0	0	1	0	2.01E-02	-0.37	Down
ENSG00000156411	*ATP5MPL*	1	0	0	0	2.10E-02	-0.69	Down
ENSG00000133740	*E2F5*	1	0	0	0	2.11E-02	-0.28	Down
ENSG00000184110	*EIF3C*	1	0	0	0	2.18E-02	-1.00	Down

ENSG00000165996	*HACD1*	0	0	1	0	2.22E-02	-0.37	Down
ENSG00000132003	*ZSWIM4*	1	0	0	0	2.22E-02	0.70	Up
ENSG00000111832	*RWDD1*	0	0	1	0	2.26E-02	-0.46	Down
ENSG00000187678	*SPRY4*	0	0	1	0	2.28E-02	0.44	Up
ENSG00000134463	*ECHDC3*	0	0	1	0	2.29E-02	-0.33	Down
ENSG00000111052	*LIN7A*	0	1	0	0	2.31E-02	-0.46	Down
ENSG00000154305	*MIA3*	1	0	0	0	2.31E-02	-0.30	Down
ENSG00000132326	*PER2*	0	0	1	0	2.31E-02	0.75	Up
ENSG00000116750	*UCHL5*	1	0	0	0	2.35E-02	-0.35	Down
ENSG00000036257	*CUL3*	1	0	0	0	2.37E-02	-0.44	Down
ENSG00000006118	*TMEM132A*	1	0	0	0	2.38E-02	0.55	Up
ENSG00000137857	*DUOX1*	0	0	1	0	2.39E-02	0.65	Up
ENSG00000142494	*SLC47A1*	0	0	0	0	2.39E-02	0.58	Up
ENSG00000066697	*MSANTD3*	1	0	0	0	2.50E-02	-0.88	Down
ENSG00000172932	*ANKRD13D*	0	0	1	0	2.50E-02	0.53	Up
ENSG00000164620	*RELL2*	1	0	0	0	2.58E-02	0.60	Up
ENSG00000186575	*NF2*	1	0	0	0	2.59E-02	-0.74	Down
ENSG00000031823	*RANBP3*	1	0	0	0	2.60E-02	-0.48	Down
ENSG00000122483	*CCDC18*	1	0	0	0	2.61E-02	-0.35	Down
ENSG00000156414	*TDRD9*	0	0	1	0	2.61E-02	-0.78	Down
ENSG00000066777	*ARFGEF1*	1	0	0	0	2.61E-02	-0.31	Down
ENSG00000117395	*EBNA1BP2*	1	0	0	0	2.62E-02	-0.66	Down
ENSG00000104368	*PLAT*	0	0	1	0	2.63E-02	0.64	Up
ENSG00000113811	*SELENOK*	0	0	0	0	2.63E-02	-0.34	Down
ENSG00000124507	*PACSIN1*	0	0	1	0	2.63E-02	-0.71	Down
ENSG00000186567	*CEACAM19*	0	0	0	0	2.63E-02	-0.60	Down
ENSG00000131469	*RPL27*	1	0	0	0	2.65E-02	-0.56	Down
ENSG00000100767	*PAPLN*	0	0	0	0	2.66E-02	0.97	Up
ENSG00000078304	*PPP2R5C*	1	0	0	0	2.67E-02	-0.41	Down
ENSG00000107242	*PIP5K1B*	0	0	1	0	2.67E-02	-0.47	Down
ENSG00000167393	*PPP2R3B*	1	0	0	0	2.67E-02	-0.33	Down
ENSG00000140937	*CDH11*	0	0	1	0	2.70E-02	0.56	Up
ENSG00000155099	*PIP4P2*	0	0	1	0	2.81E-02	-0.63	Down
ENSG00000196482	*ESRRG*	0	0	1	0	2.82E-02	-0.47	Down
ENSG00000159176	*CSRP1*	1	0	0	0	2.83E-02	0.52	Up
ENSG00000196396	*PTPN1*	1	0	0	0	2.84E-02	-0.52	Down
ENSG00000091527	*CDV3*	1	0	0	0	2.85E-02	-0.40	Down
ENSG00000159674	*SPON2*	0	0	0	0	2.85E-02	0.62	Up
ENSG00000134240	*HMGCS2*	0	0	1	0	2.89E-02	-2.05	Down
ENSG00000169155	*ZBTB43*	0	0	0	0	2.95E-02	-0.40	Down
ENSG00000164134	*NAA15*	1	0	0	0	2.98E-02	-0.41	Down
ENSG00000198356	*ASNA1*	1	0	0	0	3.01E-02	-0.78	Down
ENSG00000143158	*MPC2*	1	0	0	0	3.02E-02	-0.24	Down
ENSG00000160219	*GAB3*	1	0	0	0	3.08E-02	0.59	Up
ENSG00000250159	*AC106791.1*	1	0	0	0	3.09E-02	-0.62	Down
ENSG00000103034	*NDRG4*	0	0	0	0	3.09E-02	-0.34	Down
ENSG00000147601	*TERF1*	0	0	1	0	3.11E-02	0.42	Up
ENSG00000106034	*CPED1*	0	0	0	0	3.11E-02	0.37	Up
ENSG00000130724	*CHMP2A*	1	0	0	0	3.12E-02	-0.40	Down
ENSG00000136848	*DAB2IP*	1	0	0	0	3.13E-02	-0.50	Down
ENSG00000145592	*RPL37*	1	0	0	0	3.18E-02	-0.53	Down
ENSG00000148411	*NACC2*	0	0	1	0	3.26E-02	-0.47	Down
ENSG00000179262	*RAD23A*	0	0	0	0	3.28E-02	-0.32	Down

ENSG00000164088	*PPM1M*	0	0	1	0	3.31E-02	0.44	Up
ENSG00000169718	*DUS1L*	0	0	0	0	3.31E-02	-0.37	Down
ENSG00000196428	*TSC22D2*	0	0	1	0	3.32E-02	0.44	Up
ENSG00000204261	*PSMB8-AS1*	1	0	0	0	3.32E-02	0.39	Up
ENSG00000132688	*NES*	0	0	1	0	3.32E-02	0.28	Up
ENSG00000107736	*CDH23*	0	0	0	0	3.35E-02	0.71	Up
ENSG00000115325	*DOK1*	1	0	0	0	3.35E-02	0.48	Up
ENSG00000182636	*NDN*	0	1	0	0	3.36E-02	0.44	Up
ENSG00000125170	*DOK4*	0	0	1	0	3.38E-02	0.38	Up
ENSG00000168275	*COA6*	0	0	1	0	3.40E-02	-0.41	Down
ENSG00000108883	*EFTUD2*	0	0	0	0	3.43E-02	-0.26	Down
ENSG00000121579	*NAA50*	1	0	0	0	3.43E-02	-0.74	Down
ENSG00000093010	*COMT*	0	0	1	0	3.44E-02	0.25	Up
ENSG00000139990	*DCAF5*	0	0	0	0	3.45E-02	-0.34	Down
ENSG00000011485	*PPP5C*	1	0	0	0	3.47E-02	-0.32	Down
ENSG00000120688	*WBP4*	1	0	0	0	3.47E-02	-0.45	Down
ENSG00000019995	*ZRANB1*	0	0	0	0	3.49E-02	-0.24	Down
ENSG00000160877	*NACC1*	0	0	0	0	3.49E-02	-0.50	Down
ENSG00000138100	*TRIM54*	1	0	0	0	3.50E-02	-0.35	Down
ENSG00000175602	*CCDC85B*	0	0	0	0	3.51E-02	0.56	Up
ENSG00000005889	*ZFX*	0	0	1	0	3.52E-02	-0.34	Down
ENSG00000136279	*DBNL*	0	0	1	0	3.56E-02	-0.24	Down
ENSG00000157240	*FZD1*	1	0	0	0	3.56E-02	0.51	Up
ENSG00000137171	*KLC4*	1	0	0	0	3.58E-02	0.29	Up
ENSG00000162601	*MYSM1*	0	0	1	0	3.61E-02	-0.54	Down
ENSG00000230630	*DNM3OS*	0	0	1	0	3.63E-02	0.65	Up
ENSG00000143869	*GDF7*	0	0	0	0	3.65E-02	0.76	Up
ENSG00000141568	*FOXK2*	1	0	0	0	3.68E-02	-0.28	Down
ENSG00000090061	*CCNK*	1	0	0	0	3.69E-02	-0.25	Down
ENSG00000151725	*CENPU*	1	0	0	0	3.72E-02	0.62	Up
ENSG00000182253	*SYNM*	1	0	0	0	3.72E-02	-0.29	Down
ENSG00000263335	*AF001548.2*	1	0	0	0	3.79E-02	0.52	Up
ENSG00000103316	*CRYM*	0	0	1	0	3.80E-02	0.52	Up
ENSG00000167705	*RILP*	0	0	1	0	3.80E-02	-0.42	Down
ENSG00000071127	*WDR1*	1	0	0	0	3.88E-02	-0.46	Down
ENSG00000182287	*AP1S2*	0	0	0	0	3.90E-02	-0.34	Down
ENSG00000114670	*NEK11*	0	0	0	0	3.94E-02	0.77	Up
ENSG00000178764	*ZHX2*	1	0	0	0	3.94E-02	-0.39	Down
ENSG00000197977	*ELOVL2*	0	0	0	0	3.96E-02	-0.71	Down
ENSG00000179041	*RRS1*	1	0	0	0	4.03E-02	-0.55	Down
ENSG00000179958	*DCTPP1*	1	0	0	0	4.07E-02	-0.47	Down
ENSG00000100418	*DESI1*	0	0	1	0	4.09E-02	-0.29	Down
ENSG00000196843	*ARID5A*	0	0	1	0	4.11E-02	0.51	Up
ENSG00000168763	*CNNM3*	1	0	0	0	4.13E-02	0.37	Up
ENSG00000165487	*MICU2*	1	0	0	0	4.16E-02	-0.58	Down
ENSG00000170004	*CHD3*	0	0	0	0	4.20E-02	0.48	Up
ENSG00000127191	*TRAF2*	0	0	1	0	4.26E-02	0.46	Up
ENSG00000198873	*GRK5*	1	0	0	0	4.33E-02	-0.70	Down
ENSG00000019582	*CD74*	0	1	0	0	4.34E-02	0.47	Up
ENSG00000110925	*CSRNP2*	0	0	1	0	4.36E-02	0.19	Up
ENSG00000168615	*ADAM9*	0	0	0	0	4.38E-02	-0.39	Down
ENSG00000118762	*PKD2*	0	0	0	0	4.42E-02	0.32	Up
ENSG00000141582	*CBX4*	1	0	0	0	4.42E-02	-0.51	Down

ENSG00000171476	*HOPX*	0	0	1	0	4.45E-02	-1.37	Down
ENSG00000103260	*METRN*	0	0	1	0	4.48E-02	0.38	Up
ENSG00000078061	*ARAF*	0	0	0	0	4.54E-02	-0.27	Down
ENSG00000090776	*EFNB1*	0	0	1	0	4.54E-02	-0.33	Down
ENSG00000186716	*BCR*	1	0	0	0	4.57E-02	-0.71	Down
ENSG00000113140	*SPARC*	0	0	0	0	4.57E-02	0.49	Up
ENSG00000144645	*OSBPL10*	0	0	0	0	4.62E-02	0.85	Up
ENSG00000164683	*HEY1*	1	0	0	0	4.62E-02	0.75	Up
ENSG00000008869	*HEATR5B*	1	0	0	0	4.75E-02	-0.72	Down
ENSG00000112335	*SNX3*	1	0	0	0	4.81E-02	-0.42	Down
ENSG00000164897	*TMUB1*	1	0	0	0	4.81E-02	-0.52	Down
ENSG00000126218	*F10*	0	1	0	0	4.82E-02	0.54	Up
ENSG00000168994	*PXDC1*	1	0	0	0	4.90E-02	0.59	Up
ENSG00000136888	*ATP6V1G1*	1	0	0	0	4.91E-02	-0.49	Down
ENSG00000277196	*AC007325.2*	0	0	1	0	4.94E-02	-1.03	Down
ENSG00000003096	*KLHL13*	0	0	0	0	4.99E-02	0.83	Up

1: ‘1’ or ‘0’ indicates if the gene was identified as differentially expressed gene or not; 2: FDR-adjusted p-value from meta-analysis; 3: Average of log_2FC from individual studies; 4: ‘Up’ or ‘Down’ indicates if the gene was upregulated or downregulated. The genes were sorted based on the adjusted p-values.

Supplementary Table S2. Significant canonical pathways with absolute z-score > 2.0 and their involved genes.

**Ingenuity Canonical Pathways**	**-Log(p)[1]**	**z-score[2]**	**Genes**
EIF2 Signaling	11.9	-3.27	*ACTC1, ATF4, CCND1, EIF3C, EIF3H, EIF3K, EIF4G1, EIF5B, MT-TM, PIK3CB, PIK3CD, PPP1CC, PPP1R15A, RAP1A, RPL13A, RPL14, RPL19, RPL26, RPL27, RPL27A, RPL35, RPL37, RPL3L, RPL4, RPL7A, RPL8, RPLP0, RPLP1, RPLP2, RPS12, RPS24, RPS5, RPS7, RPS8, UBA52*
Oxidative Phosphorylation	2.52	-3.32	*ATP5F1C, ATP5F1E, ATP5MC1, COX17, COX5A, COX7B, MT-ND1, MT-ND2, NDUFA6, NDUFS5, UQCR10*
Senescence Pathway	2.02	-2.07	*ARAF, CAPN1, CCND1, E2F5, EIF4EBP1, EP300, GADD45A, KAT2B, MAPK7, MTOR, PIK3CB, PIK3CD, PPP2R3B, PPP2R5C, PPP3CC, RAP1A, SQSTM1, TGFBR2, TGFBR3*
Superpathway of Inositol Phosphate Compounds	1.4	-3.05	*ATP1A1, INPPL1, ITPK1, PIK3CB, PIK3CD, PIP4P2, PIP5K1B, PLPP7, PPIP5K2, PPP1CC, PPP1R14B, PPP5C, PTPN1*

1: -log(p) > 1.3 reflects a significant association between a canonical pathway and its involved genes; 2: Positive and negative z-scores are considered as activated and inhibited, respectively. The pathways were sorted based on the -log(p).

Supplementary Table S3. Differentially expressed upstream regulators and their targeted genes.

Upstream Regulator	Log_2FC	Molecule Type	Activation z-score[1]	p-value of overlap[2]	Target Molecules in Dataset
SMARCA4	-0.38	transcription regulator	0.47	4.61E-10	*ADGRG1, ANO1, AP1S2, ASCC1, AZGP1, BMP4, BNIP3L, C19orf12, C1orf54, CARD16, CASP1, CCDC85B, CCDC9B, CCN2, CCND1, CD74, CDH11, CKM, CORO6, CP, CTSB, CYP4B1, DES, ECM2, EFNB1, EGR1, ELOVL6, FBLIM1, FHL2, GADD45A, HEY1, IGF1, IGFBP5, LOX, LUM, MEIS1, MGP, MMP2, MN1, MT-ND2, MYH11, MYL5, NECTIN1, PLAT, PLIN2, PTP4A2, RAD1, RAMP1, SERPINE2, SLC11A1, SMARCA4, SOCS2, SOD3, SOX17, ST3GAL1, STARD10, TNNT2, TPM1, TUBA3C/TUBA3D, UBE2H*
ERBB2	-0.39	kinase	0.70	5.77E-07	*ACAA2, ADAM19, ATF4, BCL2L1, CCDC80, CCN2, CCND1, CCND2, CDC37, CDC42SE1, CDH11, CHCHD10, CTSB, CUL3, CYP2J2, CYP4B1, DAG1, DOK1, DPT, E2F5, EBP, EGR1, EIF4EBP1, EMP1, ERBB2, ESR2, F2R, FLOT2, FSCN1, GPX3, GTF3A, HEY1, IGFBP5, JAK1, JUP, KLHDC3, LTBP2, LUM, MAF, MKNK2, NDRG4, PDGFD, PGM1, PIK3CD, PLAT, PLIN2, POLR1D, PRDX2, PSMC3, PTPN1, RAB31, SLC2A1, SOCS2, SOX4, SPARC, STAT3, THBS4, TPM1, TUBA1A, UQCR10*
TBX5	-0.59	transcription regulator	-2.89	7.79E-07	*ACTC1, ATP2A2, BCL2L1, DES, ECM2, FAP, HSPB7, MYH6, NKX2-5, NPPA, PLA2G2A, SCN5A, TNNT2, TPM1, TTN*
ESR2	-0.78	ligand-dependent nuclear receptor	-0.47	9.16E-06	*ACTC1, AKAP1, APEX1, ASB2, BAG1, BCL2L1, BMP4, CCN2, CCND1, COL16A1, COMP, EGR1, EP300, ERBB2, ERN1, ESR2, FBLN1, FSCN1, IGF1, JAK1, LOX, MICOS10-NBL1/NBL1, MMP2, MYH6, MYH7, NDRG2, PLAT, PLEC, PLIN2, PLXDC1, PTH1R, S100A9, SFRP4, SLC25A4, SOCS2, SOX4, ST8SIA1, TGFBR2, TGM2, THBS4, TPM1, USP8*
CCN2	1.19	growth factor	0.88	2.08E-05	*BCL2L1, CCN2, CCND1, CFLAR, COL8A1, EGR1, IGF1, LOX, MIA3, MMP2, PTH1R, RAMP2, SFRP4, SOX4, SPARC*
AZGP1	-0.85	transporter	-1.13	3.05E-05	*AZGP1, BCL2L1, BMP4, CCND1, DSP, EGR1, MTOR*
TEAD1	-0.73	transcription regulator	0.43	7.52E-05	*CCND1, DES, FIBIN, IGFBP5, LATS2, MYH6, MYH7, NPPA, SULF1, TNNT2, TPM1*
MTA1	-0.79	transcription regulator	-0.60	8.74E-05	*ACTC1, APEX1, BCL2L1, CCND1, CCND2, EMP1, MMP2, MYH6, STAT3*
HMGA1	-0.57	transcription regulator	-0.22	1.00E-04	*CCND1, CCND2, EGR1, IER2, IGF1, ITGAL, KCNJ11, NECTIN1, NF2, NKX2-5, PTH1R, RPL7A, SOD3, SOX17, SOX4, SPARC, STAT3*
IGF2BP2	-0.73	translation regulator		2.48E-04	*ELOVL6, LIMS2, TRIM54*
SFRP4	2.62	transmembrane receptor	2.00	3.06E-04	*CCND1, MYH6, MYH7, NKX2-5, TNNT2*

STAT3	-0.91	transcription regulator	-0.04	1.11E-03	*APEX1, BCL2L1, BCL6, BCL9L, BECN1, BNIP3L, BOC, CASP1, CCN2, CCND1, CCND2, CD74, CFLAR, COPS5, CTSB, EGR1, ESR2, FSCN1, GADD45A, HEY1, IGFBP5, JAK2, KAT2B, LOX, MAF, MMP2, MT-ND1, MT-ND2, MYH7, NES, NPPA, PHLDA1, PLA2G2A, PRF1, PTN, S100A9, SERPINE2, SLC1A3, SLC2A1, STAT3*
CUL3	-0.44	enzyme	-0.82	1.14E-03	*CKMT2, DBP, GSTK1, MFAP4, MYH7, PDLIM5, S100A9, SPON1*
NKX2-5	-0.85	transcription regulator	0.02	1.27E-03	*CBY1, DIO2, HOPX, NKX2-5, NPPA, SCN5A, TBX5*
TRIM54	-0.35	other		1.60E-03	*FHL2, FLNC*
TNNT2	-0.55	other		1.60E-03	*MYH7, NPPA*
SOCS2	0.90	other		1.85E-03	*IGF1, MYH7, PIM3, STAT3*
PER2	0.76	transcription regulator		1.85E-03	*ATP1A1, CCND1, DBP, PER2*
TTN	-0.67	kinase		3.07E-03	*ANKRD2, ATP2A2, FHL2*
ERN1	0.88	kinase	-0.29	3.88E-03	*ATF4, ATP2A2, BECN1, GOT1, LAD1, MAP1LC3B, MYH7, NPPA, PLIN2, PVR, SPARC, SYT11, TINAGL1*
MTOR	-0.45	kinase	-0.08	4.06E-03	*ACACB, ATP2A2, BCL2L1, CASP1, CCND1, CFLAR, CITED2, DAG1, DDAH2, ELOB, FAF1, HADHA, HMGCS2, IGF1, JAK2, MAP1LC3B, MTOR, NCAM1, NDUFA6, PGP, PTH1R, SLC2A1, SOX4, SQSTM1, STAT3*
DAG1	-0.65	transmembrane receptor	-1.00	6.10E-03	*DAG1, SLC2A1, SNTA1, SSPN*
JAK2	0.82	kinase	0.64	6.56E-03	*BCL2L1, CASP1, CCND1, CCND2, CFLAR, EGR1, F10, JAK2, NDN, SLC11A1, SLC1A3, STAT3, TGM2*
TFRC	-1.17	transporter	0.56	6.86E-03	*ADGRG1, ATF4, GADD45A, GPX3, PIP5K1B, PPP1R15A, PTN, SLC2A1, UBE2E1*
PPP1R15A	0.72	other		7.39E-03	*ATF4, CCND1, GADD45A, MAP1LC3B*
PPP1R13L	-0.54	transcription regulator	1.07	7.39E-03	*CCND2, DSP, JUP, SLC2A1*
F2R	0.92	G-protein coupled receptor	2.56	8.24E-03	*CASP4, CCN2, CCND1, CDH11, DSP, EGR1, F2R, MMP2, PLAT, TGM2*
IGFBP5	0.48	other	1.98	1.05E-02	*BCL2L1, CCND1, IGF1, MYH7*
TEAD2	-0.51	transcription regulator	1.00	1.30E-02	*CCND1, FIBIN, IGFBP5, SULF1, TNNT2, TPM1*
HEY1	0.75	transcription regulator	-0.22	1.43E-02	*MMP2, MYH11, MYH6, NPPA*
F10	0.54	peptidase		1.67E-02	*CCN2, EGR1, MMP2*
SOX4	1.06	transcription regulator	1.01	1.77E-02	*APBB1, B3GNT5, CASP1, CD74, CTNNA1, DDAH2, EMP1, IGF1, LHFPL6, NCAM1, PRXL2A, SERPINE2, TEAD1, TUBA1A*

S100A9	-1.55	other	1.50	1.85E-02	*BECN1, CASP1, CCND1, CP, EPB41L4B, FBLIM1, FURIN, IGF1, KLF15, LOC102724788/PRODH, MMP2, PHLDA1, S100A9*
APBB1	-0.57	transcription regulator		2.03E-02	*CASP4, CCND1, TPM1*
MT-TM	-1.19	other		2.03E-02	*MT-ND1, MT-TM, MT-TQ*
BMP4	0.63	growth factor	1.14	2.05E-02	*ADGRG1, ALDH1A2, BMP4, CCND1, DIO2, HEY1, HOPX, MAFK, NCAM1, NES, SOX17, SPARC, SULF1, TBX5, TGM2*
THBS2	0.80	other		2.15E-02	*HEY1, MMP2*
EFNB1	-0.33	other		2.15E-02	*BCL2L1, CFLAR*
BNIP3L	0.49	other	0.82	2.21E-02	*BCL2L1, GADD45A, IL16, PIK3CD, STAT3, TFRC*
RAPGEF1	-0.45	other		2.42E-02	*DHX38, EFTUD2, RAP1A*
CDH11	0.56	other	-1.00	2.43E-02	*IGF1, JUP, MBD2, S1PR3*
NPPA	1.86	other	-0.66	2.73E-02	*CCND1, EGR1, FLT4, NPPA*
NF2	-0.74	other	-1.98	2.73E-02	*CCND1, GLUL, MMP2, NF2*
DIO2	1.30	enzyme		3.64E-02	*ATP2A2, B3GNT5, EGR1, KCND3, MYH7, NPPA, SLC1A3*
ALDH1A2	0.80	enzyme	0.00	3.76E-02	*CCN2, NKX2-5, NPPA, SPARC*
STC1	1.41	kinase		3.81E-02	*MTOR, STC1*
KAT2B	-0.58	transcription regulator	-0.13	3.89E-02	*CCN2, CKM, KAT2B, NPPA, TGFBR2*
RBM25	-0.38	other		4.00E-02	*BCL2L1*
RPLP0	-0.92	other		4.00E-02	*CCND1*
MPP3	-1.23	kinase		4.00E-02	*NECTIN1*
EZR	-0.32	other	0.04	4.15E-02	*ATF4, BCL2L1, EIF4EBP1, MMP2*
EIF4EBP1	-0.99	translation regulator		4.15E-02	*CCND1, CFLAR, EGR1, SLC2A1*
KLF15	-0.96	transcription regulator	-0.70	4.21E-02	*BECN1, CCN2, ESRRG, HADHA, NPPA*
BCL6	-0.54	transcription regulator	0.21	4.63E-02	*BCL2L1, BCL6, CCND1, CCND2, GADD45A, HACD1, MAF, MCM3AP, MGP, PLA2G2A, SOCS2, TRMT13, TUBA3C/TUBA3D, UBA7*
CCND2	0.48	other		4.78E-02	*CCND1, CCND2*
CAPN1	-0.41	peptidase		4.78E-02	*MMP2, PTPN1*
YBX3	-0.49	transcription regulator		4.78E-02	*CCND1, ERBB2*
EP300	-0.43	transcription regulator	-0.59	4.85E-02	*ACACB, ARID5A, BCL2L1, BCL6, CCND1, CFLAR, CKM, COMT, EGR1, EP300, EPN1, ERBB2, HMGCS2, IGF1, JUP, MAF, MYH11, NPPA, PIP5K1B, PITPNM1, PLEKHF1, RCSD1, SCN4B, TANC2, TGFBR2*
ADRA1A	-0.67	G-protein coupled receptor		4.88E-02	*EGR1, LOX, NPPA, PRRX1, STAT3*

IGF1	0.82	growth factor	0.02	4.93E-02	*BCL2L1, BMP4, CCN2, CCND1, CCND2, CFLAR, CITED2, EGR1, ESR2, GADD45A, HMGA1, IER2, IGF1, IGFBP5, IL16, MMP2, MYH11, MYH6, NCAM1, NPPA, PHLDA1, PLA2G2A, PPP3CC, PTH1R, SLC12A6, SLC2A1, YBX3*
CYP2J2	0.97	enzyme		4.96E-02	*BCL2L1, CYP2J2, NPPA*

1: Positive and negative z-scores are considered as activated and inhibited, respectively; 2: A p-value of overlap indicates whether there is a statistically significant interaction between an upstream regulator and its regulated genes. A p-value of overlap < 0.05 is considered as statistically significant. The genes were sorted based on the p-value of overlap.
